# Plant-type phytoene desaturase: Functional evaluation of structural implications

**DOI:** 10.1371/journal.pone.0187628

**Published:** 2017-11-27

**Authors:** Julian Koschmieder, Mirjam Fehling-Kaschek, Patrick Schaub, Sandro Ghisla, Anton Brausemann, Jens Timmer, Peter Beyer

**Affiliations:** 1 University of Freiburg, Faculty of Biology, Freiburg, Germany; 2 University of Freiburg, Department of Physics, Freiburg, Germany; 3 University of Konstanz, Department of Biology, Konstanz, Germany; 4 University of Freiburg, Institute for Biochemistry, Freiburg, Germany; 5 University of Freiburg, BIOSS Center for Biological Signaling Studies, Freiburg, Germany; Monash University, AUSTRALIA

## Abstract

Phytoene desaturase (PDS) is an essential plant carotenoid biosynthetic enzyme and a prominent target of certain inhibitors, such as norflurazon, acting as bleaching herbicides. PDS catalyzes the introduction of two double bonds into 15-*cis*-phytoene, yielding 9,15,9'-tri-*cis*-ζ-carotene via the intermediate 9,15-di-*cis*-phytofluene. We present the necessary data to scrutinize functional implications inferred from the recently resolved crystal structure of *Oryza sativa* PDS in a complex with norflurazon. Using dynamic mathematical modeling of reaction time courses, we support the relevance of homotetrameric assembly of the enzyme observed *in crystallo* by providing evidence for substrate channeling of the intermediate phytofluene between individual subunits at membrane surfaces. Kinetic investigations are compatible with an ordered ping-pong bi-bi kinetic mechanism in which the carotene and the quinone electron acceptor successively occupy the same catalytic site. The mutagenesis of a conserved arginine that forms a hydrogen bond with norflurazon, the latter competing with plastoquinone, corroborates the possibility of engineering herbicide resistance, however, at the expense of diminished catalytic activity. This mutagenesis also supports a “flavin only” mechanism of carotene desaturation not requiring charged residues in the active site. Evidence for the role of the central 15-*cis* double bond of phytoene in determining regio-specificity of carotene desaturation is presented.

## Introduction

**Fig 1:**
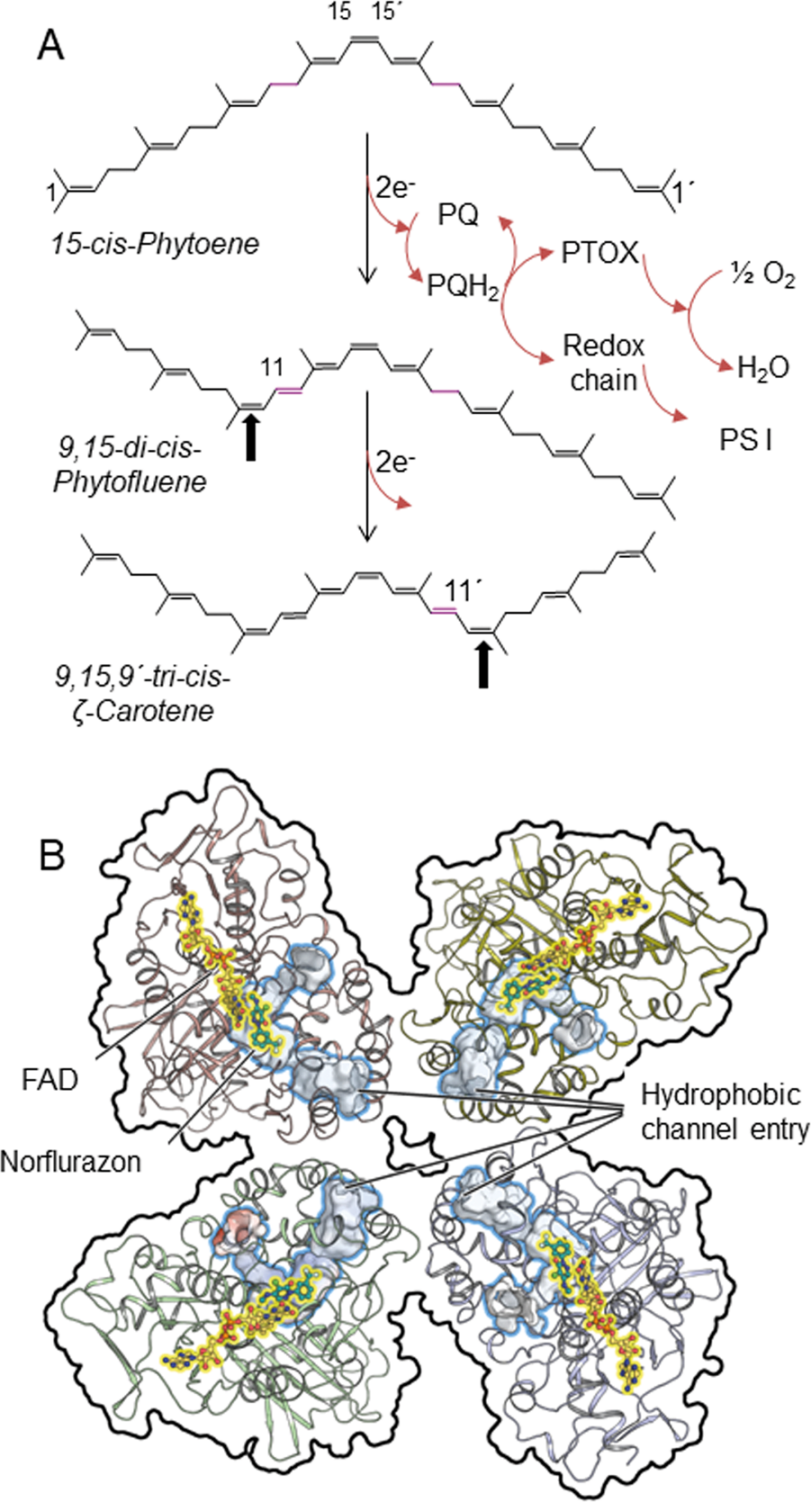
PDS reaction and structure. (A) The symmetrical substrate, 15-*cis*-phytoene is desaturated twice at the symmetrical positions indicated in magenta. The simultaneous isomerization of the adjacent double bonds (arrows) from *trans* to *cis* yields the symmetric product 9,15,9'-tri-*cis*-ζ-carotene via the asymmetric intermediate, 9,15-di-*cis*-phytofluene. Electrons are transferred from the reduced enzyme-bound FAD onto the terminal electron acceptor plastoquinone which is reoxidized by the photosynthetic electron transport chain or, alternatively, by the plastid terminal oxidase PTOX (sequence omitted in the second partial reaction). (B) Overview on the tetrameric PDS assembly as viewed from the plane of the membrane. The substrate entry channels are outlined in blue, FAD is represented as sticks and balls and highlighted in yellow, norflurazon is represented as green sticks.

Plant carotenoids are typically C_40_ isoprenoids characterized by an undecaene chromophore conferring a yellow to orange color. They are essential pigments, due to their indispensable functions as anti-oxidants, as light-harvesting photosynthetic pigments [1] and as phytohormone precursors [2] [3]. Due to the very high lipophilicity of intermediates and products, their biosynthesis takes place in membrane-associated micro-topologies within plastids. The enzyme phytoene synthase (PSY) catalyzes the first committed step by condensing two molecules of geranylgeranyl-diphosphate to yield15-*cis*-phytoene. Hereafter, phytoene desaturase (PDS)–the subject of this work–represents the entry point into the so-called poly-*cis* pathway of carotene desaturation in cyanobacteria and plants that involves a series of specific poly-*cis* configured desaturation intermediates. PDS introduces two *trans*-configured double bonds at positions C11-C12 and C11’-C12’ into the symmetric substrate phytoene (Fig 1A) and, simultaneously and obligatorily, a trans-to-*cis*-isomerization takes place at positions C9-C10 and C9’-C10’. Thus, PDS exclusively yields 9,15-di-*cis*-phytofluene as intermediate and 9,15,9’-tri-*cis*-ζ-carotene as the end product [4]. Because of the symmetry of educt and final product, the PDS reaction can formally be viewed as consisting of two identical reactions taking place at the both ends of phytoene (Fig 1A). The colorless triene chromophore of phytoene is thereby extended to a heptaene in ζ-carotene, providing a slightly yellow color. Subsequent desaturation, isomerization, cyclization and oxygenation reactions finally yield the typical complements of plant xanthophylls (for a review on the carotenoid biosynthesis pathway, see [5]).

As with many other membrane-associated proteins, PDS proved to be notoriously difficult to deal with experimentally. Purification in native state and concomitant development of conditions to maintain adequate enzymatic activity with its highly lipophilic substrates have not been satisfactorily achieved so that radiolabeled tracers needed to be employed with complex *in vitro* systems. This hampered detailed structural and mechanistic investigations. We have recently introduced a biphasic incubation system containing substrates incorporated within liposomal membranes that resulted in unprecedented photometrically detectable desaturation activity with purified rice PDS-His_6_ [6]. This experimental setup was found to work with several enzymes of this pathway [7–10]).

PDS-His_6_ from *Oryza sativa* (OsPDS-His_6_) can be purified as soluble protein. The enzyme attaches to liposomes spontaneously and converts phytoene into phytofluene and ζ-carotene in the presence of benzoquinones, all of which are incorporated into lipid phase. This behavior was interpreted as a monotopic membrane interaction. Confirming previous results [11, 12], the purified enzyme contained non-covalently bound FAD. The cofactor, being reduced upon carotene desaturation, can be reoxidized by the direct interaction with benzoquinones but not by molecular oxygen [6]. In line with this, PDS activity relies on plastoquinone in isolated chromoplasts [13] and *in planta* [14] and is thus controlled by the redox state of the plastoquinone pool, i.e. the activity of the photosynthetic electron transport chain and/or the plastid terminal oxidase PTOX (for review, see [15]). Gel permeation chromatography and electron microscopy of PDS-His_6 _in combination with incubation experiments suggested homotetramers as the minimal catalytically active and flavinylated unit while monomer fractions lose the cofactor and are inactive [6].

These advances enabled the recent elucidation of the OsPDS-His_6_ structure in a complex with its long-known inhibitor norflurazon [16]. Due to its extreme lipophilicity and length (C_40_H_64_), the co-crystallization and crystal soaking with the carotene substrate was not possible. Thus, structure-function relations were necessarily inferred from the structure, such as the suggestion of an ordered ping-pong bi-bi (S1 Fig) kinetic mechanism involving the carotene substrate and the quinone co-substrate: The tertiary structure is characterized by a single elongated, highly hydrophobic substrate cavity with its entrance located in the lipid bilayer. It provides access to the active site in proximity to the FAD flavin moiety for both long-chain substrates, the carotene and plastoquinone, which cannot occupy the cavity simultaneously (Fig 1B). Thus, carotene desaturation and flavin reoxidation by plastoquinone are envisioned as distinct events. Moreover, the length of the substrate cavity implies that the substrates are entirely accommodated therein. Norflurazon, interpreted as a quinone-analog, is coordinated via its keto group by the imino function of the conserved residue Arg300. The specific role of Arg300 in norflurazon binding is confirmed by the finding that mutations of homologous arginine residues confer resistance in cyanobacteria [17, 18] and plants [19]. In *crystallo*, PDS forms homotetramers (Fig 1B) in which the substrate channels point to each other. Intuitively, this suggests a succession of two individual and successively occurring desaturation reactions at the two identical ends of phytoene. Within the homotetramer, phytofluene might be expelled from one subunit after the first desaturation and channeled into an adjacent, oxidized subunit for the second desaturation at the saturated half side. Plastoquinone enters the cavity for flavin reoxidation after each desaturation.

The present work represents necessary functional companion work to scrutinize implications derived from the PDS structure. We have focused on those that most evidently required clarification. This pertains to (i) the potential relevance of the tetrameric assembly possibly mediating subunit cooperation. Furthermore, (ii) we provide evidence in favor of the proposed ping-pong mechanism, (iii) shed light on the mode of inhibition by norflurazon and on the role of the conserved Arg300 and (iv) address the question as to how regio-specificity of carotene desaturation is achieved.

## Materials and methods

### PDS-HIS_6_ cloning, mutagenesis, expression and purification

Rice *PDS* (Acc. AF049356) deprived of a stretch of nucleotides coding for the 87 aa transit sequence (corresponding to UniProtKB Acc. A2XDA1.2) was synthesized (Genescript) equipped with a 5' *Nde*I site and 3' *His*_*6*_ coding sequence followed by *Hind*III site. Expression vector cloning, protein expression in *E*. *coli* and purification of the protein was done as given previously [6]. Proteins were quantified using a Nanodrop photometer (Implen) with ε_280nm_ = 72,400 l mol^-1^ cm^-1^ for PDS, as estimated using the Vector NTI suite software (Invitrogen). Protein purity was routinely analyzed by SDS-PAGE on 12% polyacrylamide gels. GPC analysis of purified OsPDS-His_6_ was performed according to procedures detailed in [6].

PDS mutants were generated by overlap extension PCR [20]. The complementary primers carrying the mutations (bold) were 5' cctgaagaaa**tgt**gtttaaagcaa 3' and 5'ttgctttaaac**aca**tttcttcagg 3'(Arg300Thr), 5'cctgaagaaa**act**gtttaaagcaa 3' and 5'ttgctttaaac**agt**tttcttcagg 3' (Arg300Ser), 5'catcgaagc**gaa**atatttctgct3' and 5'agcagaaatat**ttc**gcttcgatg3' (Leu538Phe), 5' catcgaagc**cct**atatttctgc3' and 5'gcagaaatat**agg**gcttcgatg3' (Leu538Arg), 5’gggataagctcc**aac**aaagatatg3’ and 5’catatcttt**gtt**ggagcttatccc3’ (Phe162Val). The flanking primers used to generate the full length product included the *Nde*I and *Hind*III restriction sites (bold) used for insertion into *pRice-PDSHis*_*6*_ and were 5'acaaggaccatag**catatg**gct 3' and 5'acggccagtgcc**aagctt**ca3'. The mutations Tyr506Phe and Thr508Val were introduced by custom synthesis (Genescript) and inserted into *pRice-PDSHis*_*6*_ via *NdeI* and *HindIII* restriction sites.

### Liposome preparation and evaluation

Phytoene was extracted and purified from phytoene-accumulating *Escherichia coli* cells [7]. 9,15-di-*cis*-phytofluene was extracted and purified from *tangerine* tomato fruit (see carotene analysis and purification). After purification, 15-*cis*-phytoene and 9,15-di-*cis*-phytofluene concentrations were determined photometrically in hexane solution using ε_285 nm_ = 68,500 mol^-1^ l^-1^ cm^-1^ and 73,300 mol^-1^ l^-1^ cm^-1^, respectively. For liposome preparation, 5 mg phosphatidylcholine was dissolved in CHCl_3_ and added to variable amounts (50 nmol under standard assays conditions) of either phytoene or phytofluene, and dried under a stream of N_2_. After vortexing, the lipid-phytoene mixture was dried under N_2_ and 1 ml liposome-buffer (50 mM Tris-HCl, pH 8, 100 mM NaCl) was added followed by 30 min incubation on ice. Liposomes were formed by gentle sonication. Small unilamellar vesicles were formed by a passage through a French Press at 20,000 psi [21]. Phytoene and phytofluene concentrations in liposomes were verified after re-extraction using HPLC system 1 (see carotene analysis and purification).

### Enzyme assays with purified OsPDS-His_6_

The standard enzyme assay contained in a final volume of 700 μl 50 mM MES-KOH pH 6.0, 100 mM NaCl, 25 μg affinity-purified PDS-His_6_ (0.63 μM), 19.25 mM DPQ (c_eff;_ see below) and 100 μl of liposomes (0.5 mg soybean phosphatidylcholine) 10 mM phytoene (c_eff_). The liposomes in 100 μl were first supplemented with DPQ, vortexed, the buffer was added, followed by protein. The incubation was carried out at 37°C in the dark for 10 min and the reaction was stopped by addition of one equivalent volume of CHCl_3_ /MeOH (2:1, v/v).

### Analysis and purification of carotenes

PDS enzyme assays: Carotenes were extracted from PDS-His_6_ assays with CHCl_3_/MeOH (2:1,v/v). Extracts were supplemented with an external standard of either 0.3 mM α-tocopherol acetate (Sigma) or 1.25 μg ml^-1^ (final concentration) of the lipophilic organic compound VIS682A (QCR Solutions Corp). After centrifugation (20,000 x g, 5 min), the organic phase was transferred and dried using a vacuum concentrator (Eppendorf, Germany). Carotenoids were dissolved in 40 μl CHCl_3_ and analyzed by HPLC using a Prominence UFLC XR system equipped with a SPD-M20A PDA detector (Shimadzu). HPLC 1 system was used to analyze the carotene products formed. A C_30_ RP column (150 x 3 mm i.d., 5 μm; YMC) was used with the solvent system A: MeOH/tert-butylmethylether (TBME) (1:3, v/v) and B: MeOH/TBME/water (5:1:1, v/v/v). The program was developed starting with 60% A, followed by a linear gradient to 100% A within 10 min; the final conditions were maintained for 4 min.

*Dunaliella salina*: Pellets from norflurazon-treated *Dunaliella salina* (kindly provided by U. Pick, Rehovot, Israel) were sonicated in acetone for 5 min and centrifuged at 3,200 x g for 5 min. This was repeated to complete discoloration. The supernatants were combined and 10 ml petroleum ether: diethyl ether (2:1, v/v) were added. Water was added for separation and carotenes were allowed to partition into the ether phase. HPLC system 2 was used to identify the phytofluene isomers present. A C_30_ column (150 x 3 mm i.d., 5 μm; YMC) was used with the solvent-system A: MeOH/TBME (4:1, v/v) and B: MeOH/TBME/water (5:1:1, v/v/v). The gradient started with 50% A followed by a linear gradient to 60% A within 20 min and to 100% A within 5 min. Final conditions were maintained for 5 min, all at a flow rate of 0.7 ml min^-1^. This program was also used for separating phytofluene isomers from extracts of *tangerine* tomato fruits and PDS assays.

*Tangerine* tomato fruit: Fruits of the *tangerine* tomato mutant defective in the carotene *cis-trans* isomerase CRTISO [8, 22, 23] were extracted with acetone and the carotenes partitioned against petroleum ether:diethyl ether (2:1, v/v), after the addition of water to achieve phase separation. The organic phase was dried in a Rotavapor-R (Büchi). For the preparative isolation of phytofluene isomers, HPLC system 3 was used employing a preparative YMC C_30_ column (250 x 10 mm i.d., 5 μm; YMC). The column was developed isocratically with MeOH/TBME (4:1, v/v) at a flow rate of 2.2 ml min^-1^.

Daffodil chromoplasts: Chromoplasts were isolated from *Narcissus pseudonarcissus* flowers [24] and were extracted as given for *tangerine* tomato fruit. For carotenoid separation, HPLC system 4 was used. A Pack Pro C_18_ column (150 x 3 mm i.d., 3 μm; YMC) was developed isocratically with 100% acetonitrile at a flow rate of 1.2 ml min^-1^.

### LC-MS analysis of desaturation products formed from 15-cis-nor-phytoene

PDS desaturation products originating from 15-*cis*-nor-phytoene (15-*cis*-1',2',3',16',17'-penta-nor-phytoene) were identified by LC-MS using a Dionex UltiMate 3000 UPLC coupled to a Q-Exactive mass spectrometer (Thermo Fisher Scientific). Sample separation was achieved with a YMC carotenoid C30 column (150 mm x 3 mm, 5 μm; YMC) with the solvent system A: methanol / TBME / water (5:1:1, v/v/v) in 0.1% (v/v) formic acid and B: methanol / TBME (1:1, v/v) in 0.1% (v/v) formic acid. Conditions started at 50% B, increased linearly to 60% B within 15 min and to 100% B within further 5 min. Final conditions were maintained for 10 min, all at a flow-rate of 0.6 ml min^-1^. Ionization of apocarotenoids was achieved with atmospheric pressure chemical ionization (APCI) and analyzed in the positive mode. Nitrogen was used as sheath and auxiliary gas, set to 20 and 10 arbitrary units, respectively. The vaporizer temperature was set to 350°C and the capillary temperature was 320°C. The spray voltage was set to 5 kV and the normalized collision energy (NCE) to 35 arbitrary units. For data analysis the TraceFinder (3.1) software and authentic apocarotenoid standards were used.

### Quantification and determination of the effective concentrations (c_eff_) of carotenes, quinones and norflurazon in liposomal assays

Quantification: Peaks areas integrated at their individual λ_max_ were corrected according to the recovery of the internal standard and normalized according to individual molar extinction coefficients (ε_285 nm_ = 68,125 mol^-1^ l^-1^ cm^-1^; phytofluene: ε_350 nm_ = 73,300 mol^-1^ l^-1^ cm^-1^; ζ-carotene: ε_400 nm_ = 138,000 mol^-1^ l^-1^ cm^-1^). Finally, amounts were calculated using the detector response factors determined with a β-carotene standard curve. Quinones and norflurazon were quantified by HPLC using calibration curves obtained with the authentic compounds.

Determination of effective liposomal concentrations (c_eff_): A biphasic liposomal assay system was used to incorporate the lipophilic substrates phytoene and decylplastoquinone as well as the hydrophobic inhibitor norflurazon. In such assay systems the substrate and inhibitor concentrations should refer to their actual concentration within the partial specific volume of the lipid bilayer. Due to the extreme lipophilicity carotenes, the incorporation of phytoene during liposome formation was close to 100%. Being less lipophilic, the partition of NFZ and DPQ into the lipid phase was determined experimentally by pentane washing of liposomes [25]. For this purpose, 250 μl of liposome suspension were supplemented with different concentrations of NFZ and DPQ (1.5 μl from acetone and methanol stocks, respectively), mixed and allowed to partition for 10 min. Samples were split in two 100 μl aliquots and supplemented with 600 μl assay buffer (see enzyme assays). One aliquot was treated with 700 μl pentane to remove free of NFZ or DPQ. After centrifugation, the pentane-phase was removed and the aqueous phase extracted with 700 μl CHCl_3_:MeOH (2:1, v/v). The second aliquot was extracted directly with 700 μl CHCl_3_:MeOH (2:1, v/v). The organic extracts of washed and non-washed liposome samples were analyzed by HPLC. Norflurazon was detected using a YMC Pack Pro C_18_ column (150 x 3 mm i.d., 3 μm, YMC) and an isocratic flow of 0.7 ml min^-1^ of MeOH:H_2_O (1/1; v/v). DPQ was detected using HPLC system 1. Partitioning of NFZ and DPQ was linear within the concentration range of added compounds and incorporation efficiencies into the liposomes of 55% for DPQ and 86% for NFZ were estimated. The concentration of carotenes, DPQ and NFZ within the lipid bilayer refers to the lipid partial specific volume. Each assay contains 0.5 mg of phosphatidylcholine with a partial specific volume of 0.997 ml g^-1^ [26], i.e. each assay contains 0.5 μl of lipid phase (see liposome preparation and enzyme assays). The resulting concentrations of the given lipophilics within the lipid bilayer can thus be calculated. This is termed effective liposomal concentration c_eff._ and used throughout.

### Software and equations

Data from kinetic studies were fitted using the software programs VisualEnzymics and GraphPad Prism with the following equations:

Dibasic pH equation: $v=\frac{C}{\left(1+(\frac{\left[H^{+}\right]}{K_{1}})+(\frac{K_{2}}{\left[H^{+}\right]})\right)}$; Michaelis-Menten: $v=\frac{v_{max}*[S]}{K_{m}+[S]}$

Competitive inhibition: $v=\frac{v_{max}*[S]}{\left(K_{m}*(\frac{1+[I]}{K_{i}}))+[S\right]}$

Protein sequence alignments were performed with Geneious. The PDS protein crystal structure was visualized using PyMOL.

### Mathematical modeling of PDS reaction time courses and kinetics

General procedures: The model consists of a set of ordinary differential equations (ODEs) that are derived for the contributing processes following mass action kinetics. The maximum likelihood method is used to estimate model parameters such that the model prediction optimally describes the observed time resolved data. Setting up the likelihood, normally distributed noise is assumed. The cost function $\chi^{2}(\theta )=\sum_{i}\frac{(x_{i}-x(t_{i},\theta ){)}^{2}}{\sigma_{i}^{2}}$ needs to be minimized in order to maximize the likelihood. Here, *θ* denotes the model parameters, the index *i* runs over the data points taken at time *t*_*i*_ with value *x*_*i*_ and uncertainty *σ*_*i*_ and *x*(*t*_*i*_,*θ*) is the model prediction at time *t*_*i*_. The nonlinear minimization of the cost function is performed by a trust region optimizer [27]. Derivatives of the cost function, upon which the optimizer relies, are provided by sensitivity equations. Prior knowledge about parameter values, e.g. values of the initial states, are incorporated by either fixing the parameter value or adding a penalty to the cost function via a quadratic prior function. In general, the cost function can have several local optima, besides the global optimum. In order to find the global optimum a multistart approach is performed by seeding the optimization in different points of the parameter space. The ODEs and sensitivity equations are integrated with the lsodes solver [28]. Identifiability of the parameters and their confidence intervals are determined by the profile likelihood method [29]. The model was implemented using the dMod package for dynamic modeling in R [30].

Data preprocessing: For PDS reaction time courses of the conversion of the substrates phytoene and phytofluene, the amounts of phytoene, phytofluene and ζ-carotene were measured over time. The experiments were conducted in triplicate. Uncertainties for the computed mean values were first estimated by a maximum likelihood method combining the empirical mean values and variances with an error model. However, additional fluctuations between neighboring time points, larger than those represented by the replicates, were observed. They cannot be captured by the error model described above, but would lead to an underestimation of the derived parameter profiles and uncertainties. Therefore, the uncertainty parameters of the error model were estimated together with the other model parameters, including the log(*σ*^2^)-term originally contained in the log-likelihood, giving rise to the new cost function:

$$-2\;\log L\;(\theta )=\sum_{i}{\left(\frac{x_{i}(\theta )-x_{i}^{D}}{\sigma_{i}(\theta )}\right)}^{2}+\log\;\left(\sigma_{i}{\left(\theta \right)}^{2}\right)$$

The uncertainty parameters *σ*_*i*_ include a relative and an absolute contribution for each observable, e.g. $\sigma_{\left[p\right]}=\sigma_{\left[p\right]}^{rel}∙[p]+\sigma_{\left[p\right]}^{abs}$ and may vary between the different reaction time courses.

The relative normalizations of the phytoene, phytofluene and ζ-carotene measurements were investigated by a preceding optimization. It is based on conservation of mass, i.e. the total sum of carotenes is conserved over each reaction time course. Such normalization is needed because of inaccuracies during carotene quantification. The molar extinction coefficient is known for 15-*cis*-phytoene but not for 9,15-di-*cis*-phytofluene and 9,15,9’-tri-*cis*-ζ-carotene. Therefore, the molar extinction coefficients for the all-*trans* species of phytofluene and ζ-carotene are used in an approximation. Scaling parameters ${\hat{s}}_{p},{\hat{s}}_{pf}$ and ${\hat{s}}_{z}$ for phytoene, phytofluene and ζ-carotene, respectively, were estimated by minimizing the discrepancy ${\hat{s}}_{p}∙{\left[p\right]}_{t=t_{i}}+{\hat{s}}_{pf}∙{\left[pf\right]}_{t=t_{i}}+{\hat{s}}_{z}∙[z{]}_{t=t_{i}}-c$ at all time points *t*_*i*_ for an arbitrary constant *c*. Since the absolute scale incorporated by the constant *c* is unknown, the ratios $l_{1}=\frac{{\hat{s}}_{1}}{{\hat{s}}_{3}}$ and ratios $l_{2}=\frac{{\hat{s}}_{2}}{{\hat{s}}_{3}}$ including their confidence intervals are estimated by a least squares approach. The scaling parameters *s*_*p*_, *s*_*pf*_ and *s*_*z*_ used for phytoene, phytofluene and ζ-carotene in the model prediction are related to the ratios via *s*_*p*_ = *l*_2_ ⋅ *s*_*pf*_ and $s_{z}=\frac{l_{1}}{l_{2}}s_{pf}$ and the constraints on *l*_1_ and *l*_2_ are added via a quadratic prior to the cost function. For additional information about data preprocessing, see S2 Appendix.

## Results and discussion

### Basic characterization of PDS in a biphasic assay system

**Fig 2:**
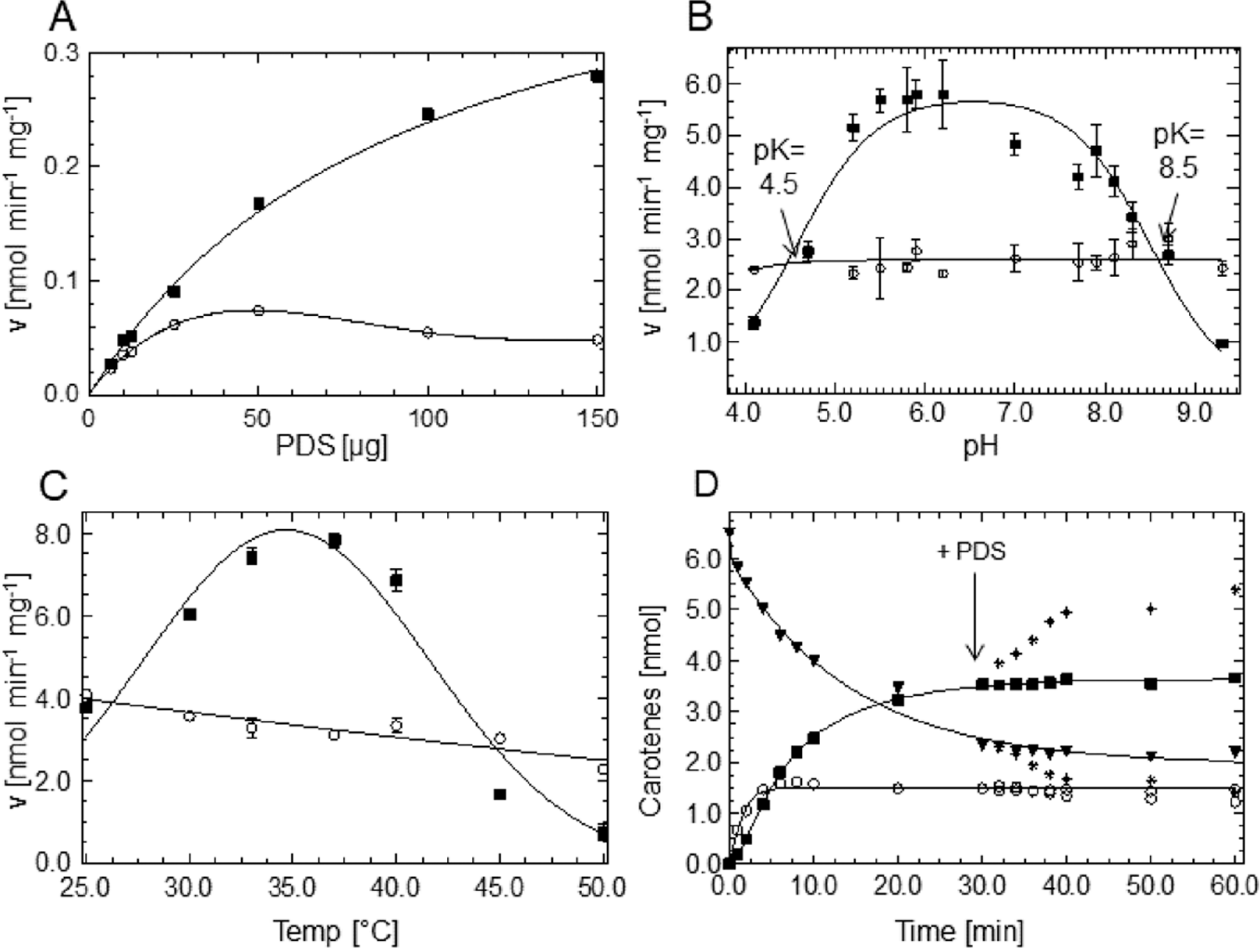
Basic characterization of the PDS reaction. Dependency of the PDS reaction rate on protein concentration (A), pH (B) and temperature (C) and reaction time course of phytofluene and ζ-carotene formation from phytoene (D). ▼, phytoene; ○, phytofluene; ■, ζ-carotene. Each experiment (A-C) was carried out using the optimum values of the respective non-variable parameters e.g. pH 6.0, 37°C in A, etc. The optimal values obtained defined the standard incubation conditions (see Methods). The standard protein concentration was set to 25 μg PDS per assay. [p] = 10 mM, [DPQ] = 19.25 mM, as determined elsewhere (see Fig 6). The samples were analyzed by HPLC after an incubation time of 10 min. Data represent the mean of duplicates (A, C) or triplicates (B) ± SEM. D, Asterisks denote the activation of phytofluene and ζ-carotene formation upon the addition of fresh PDS during the plateau phase after 30 min. Data were fitted with splines in A, C and D and with the dibasic pH equation (see Methods) in B.

**Fig 3:**
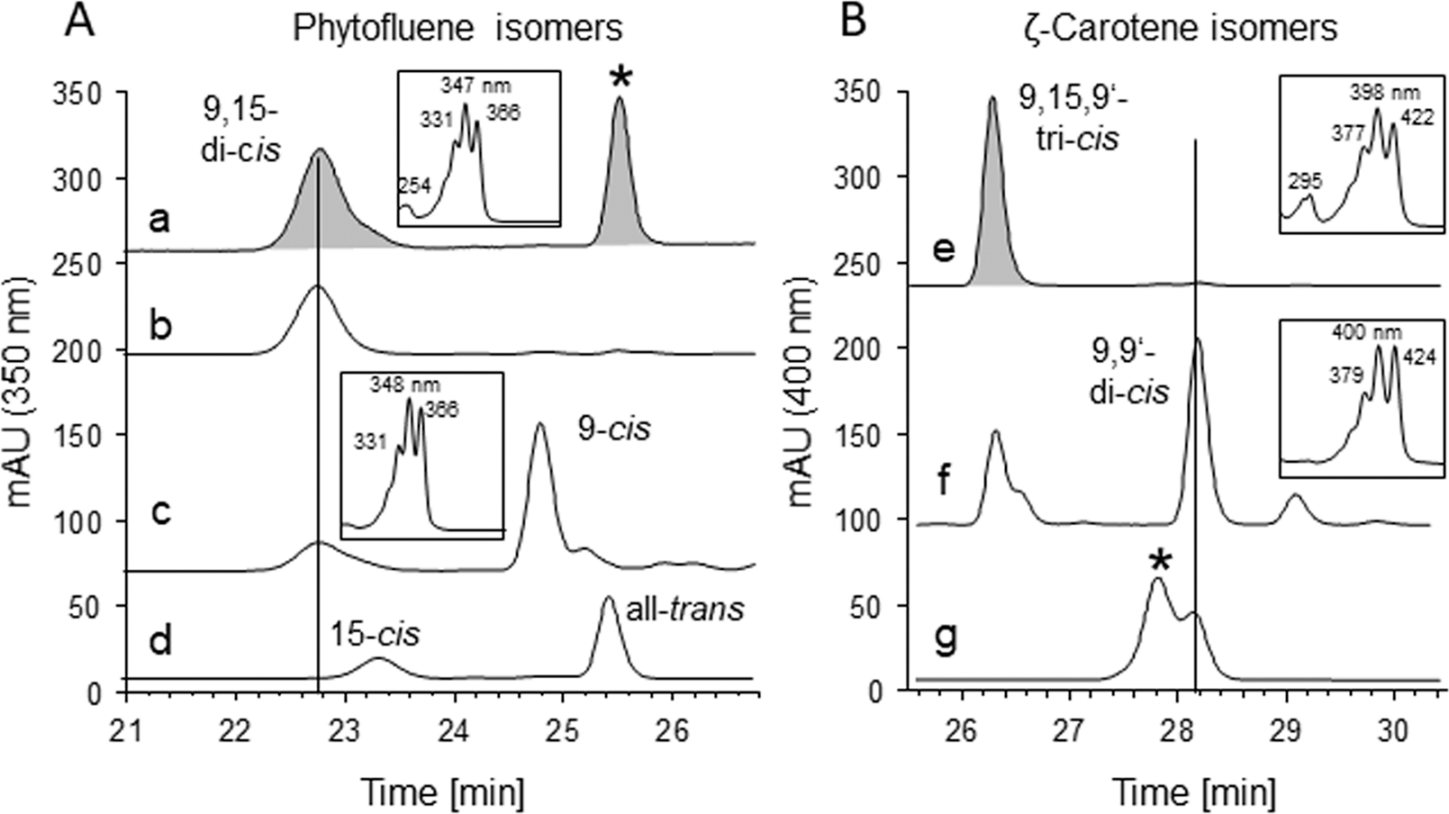
Stereoconfiguration of PDS products. (A) Phytofluene isomers: trace a represents phytofluene from a PDS assay. The peak marked with * represents the ζ-carotene formed. Only the correct 9,15-di-*cis*-phytofluene isomer is formed as revealed by comparison with authentic standards isolated from sources where *cis*-configurations are known, such as trace b, phytofluene from the *tangerine* mutant of tomato fruit [31] and trace c, phytofluene from *Dunaliella bardawil* grown in the presence of norflurazon [32]. The synthetic standards all-*trans* and 15-*cis*-phytofluene are shown in trace d. (B) ζ-carotene isomers: trace e, from PDS assays. Only the correct 9,15,9’-tri-*cis-*ζ-carotene is formed, as revealed by the effect of illumination of the PDS assay (trace f) whereby the photolabile central double bond is isomerized to *trans* [4, 24] yielding the 9,9’-di-*cis* species accompanied by small amounts of the 9-*cis* and all-*trans* species. Trace g, extract from *tangerine* tomato fruit containing 9,9’-di-*cis*-ζ-carotene. The peak marked with * represents β-carotene, detected because of spectral overlap. HPLC traces (HPLC system 2) were recorded at 400 nm. UV/VIS spectra are given as insets.

Using the biphasic liposome-based assay established for PDS-His_6_ [6], the dependence of the PDS reaction rates on protein concentration, pH and temperature was determined under standard conditions, using effective liposomal substrate concentrations c_eff_ (see Methods). Optimal pH and temperature conditions for the formation of the final product, ζ-carotene can be identified and increasing protein concentrations show to be progressively favorable for end product formation (Fig 2). In contrast, the intermediate phytofluene is barely responding to these variables, this leading to varying product:intermediate ratios. The reason may reside in unspecific isomerization of the correct 9,15-di-*cis*-phytofluene isomer giving rise to species with inappropriate stereo-configurations *in vitro*. These would not be converted because of the known strict stereospecificity of the PDS reaction (Fig 1A). However, HPLC analysis revealed that the stereo-configuration of phytofluene was correct (Fig 3A). The ζ-carotene formed was also in the correct 9,15,9’-tri-*cis-*configuration (Fig 3B), as confirmed by its photoisomerization into 9,9'-di-*cis*-ζ-carotene and its enzymatic desaturation into prolycopene (7’,9’,9,7-tetra-*cis*-lycopene; S2 Fig). Thus, the PDS reaction maintains stereo-specificity *in vitro*. Alternatively, the released intermediate may represent a steady state situation: The release of the intermediate phytofluene indicates that the two formally identical desaturation reactions might represent distinct processes that are kinetically inequivalent.

PDS requires plastoquinone as a directly interacting co-substrate to reoxidize the enzyme-bound FAD_red_ formed upon desaturation [6]. The enzyme structure has led to the conclusion that both lipophilic substrates are bound inside the same substrate cavity that cannot be occupied by both simultaneously. Thus, an ordered ping-pong bi-bi mechanism has been proposed for the sequence of kinetic events [16] (S1 Fig). In support of this, the desaturation reaction *per se* shows to be independent of DPQ: In incubations carried out under standard conditions but in the *absence* of DPQ, 1.25 nmol of flavinylated PDS monomers led to the formation of 0.66 ± 0.01 nmol phytofluene and 0.61 ± 0.14 nmol ζ-carotene. This equals 1.88 nmol of double bonds formed which is in the range of the protein amount used. Consequently, each monomer likely introduces one single double bond, i.e. carries out one carotene desaturation reaction, in the absence of the quinone. Thus, DPQ is only required to reoxidize the flavin in a separate event to enable repeated cycles of desaturation. Thus, the redox reactions between phytoene (or phytofluene) and FAD_ox_, are thermodynamically favored. The dependence of PDS on the redox state of the plastoquinone pool [33] should therefore be viewed in the context of FAD_ox_ regeneration, this being mandatory for repeated catalytic cycles.

Taken at face value, reaction time courses of PDS (Fig 2D) suggest a situation corresponding to an approach to equilibrium in which ca. 50% of the end product ζ-carotene are formed reversibly. The extent of product formation would be governed by the thermodynamics of the redox and isomerization processes in PDS (as for CRTISO in [8]). This interpretation would, however, be in contrast to the arguments outlined above. Moreover, mathematical modeling (see below) indicated that the plateau is caused by enzyme inactivation. In fact, the addition of fresh enzyme (arrow in Fig 2D) allows a resurgence of product formation in the standard assay (containing an excess of DPQ) that can lead up to > 95% of the end product, ζ-carotene. Moreover, attempts to reverse the reaction, i.e. to saturate ζ-carotene and phytofluene in the presence of DPQH_2_ (produced by the NADH-dependent reduction of DPQ by DT diaphorase [10]) were not successful as no formation of saturated carotenes was detectable. Based on these experiments and since the thermodynamic equilibrium should not be affected by subsequent additions of active enzyme, it was concluded that progressive enzyme inactivation—frequently encountered as an artifact with highly purified proteins—is the cause of incomplete substrate conversion.

### Dynamic modeling of the PDS reaction time course suggests relevance of homotetrameric assemblies

PDS shows homotetrameric assembly *in crystallo* with the substrate channels pointing towards each other (Fig 1) and the active center structure suggests an ordered ping-pong bi-bi mechanism (see Introduction, S1 Fig and [16]). The individual monomers can be regarded as bifunctional phytoene and phytofluene desaturases. The two formally identical desaturation reactions would occur in strict consecutive order and depend on each other kinetically, like in two-enzyme cascades, with phytoene and phytofluene competing for the enzyme [34]. Provided that the tetramer is also present at membrane surfaces, it would be intuitive to assume a channeling of phytoene between two of the four adjacent enzyme subunits, each introducing one double bond into the opposite identical half sides. Regarding kinetics, this would be equivalent to substrate channeling of the intermediary phytofluene between two subunits within a PDS homotetramer. However, the observation that phytofluene is released from the enzyme (Fig 2), i.e. that phytoene and phytofluene are in competition at the active site, indicates that this assumption may not, or may only partially apply. Alternatively, the intermediary phytofluene is expelled into the membrane where it diffuses to eventually be bound by its saturated end by any oxidized subunit of the same or a different homotetramer.

We have resorted to dynamic mathematical modeling of PDS reaction time courses to disentangle these two scenarios that cannot be distinguished experimentally. Three reaction time courses were used for this purpose of which two were conducted at different initial phytoene concentrations (p high, p low) and one was conducted with phytofluene as the substrate (pf). The aim was to define one set of rate constants able to describe all three reaction time courses (simultaneous parameter estimation). Doing so, the substrate channeling scenario was challenged by assuming the contrary, i.e. that PDS monomers (within the tetramer) acted individually. For modeling, the following fundamental processes are assumed to be mechanistically independent of each other (although being kinetically dependent): (i) the desaturation of phytoene (p) to phytofluene (pf), (ii) the desaturation of phytofluene (pf) to ζ-carotene (z) and (iii) the reoxidation of FAD_red_ formed during (i) and (ii) by the terminal acceptor DPQ (Q).

In an initial model, each of the major processes (i—iii) consists of three sub-processes, representing equilibria, and including all forward and reverse reactions into the mathematical model requires 18 rate constants, i.e. parameters. Details on this initial model are given in S1 Appendix. Briefly, one might expect that the large number of rate constants of this model would provide enough freedom to describe all three reaction time courses simultaneously. However, it failed to describe the observed plateaus of pf and z formation with simultaneous parameter estimation for the two reaction time courses of phytoene. Thus, a fundamental process was missing which showed to be the stagnation of PDS activity caused by inactivation. Implementing enzyme inactivation into the model allowed describing the data, however, the model was overparameterized, i.e. not all parameters could be determined with the available data.

**Fig 4:**
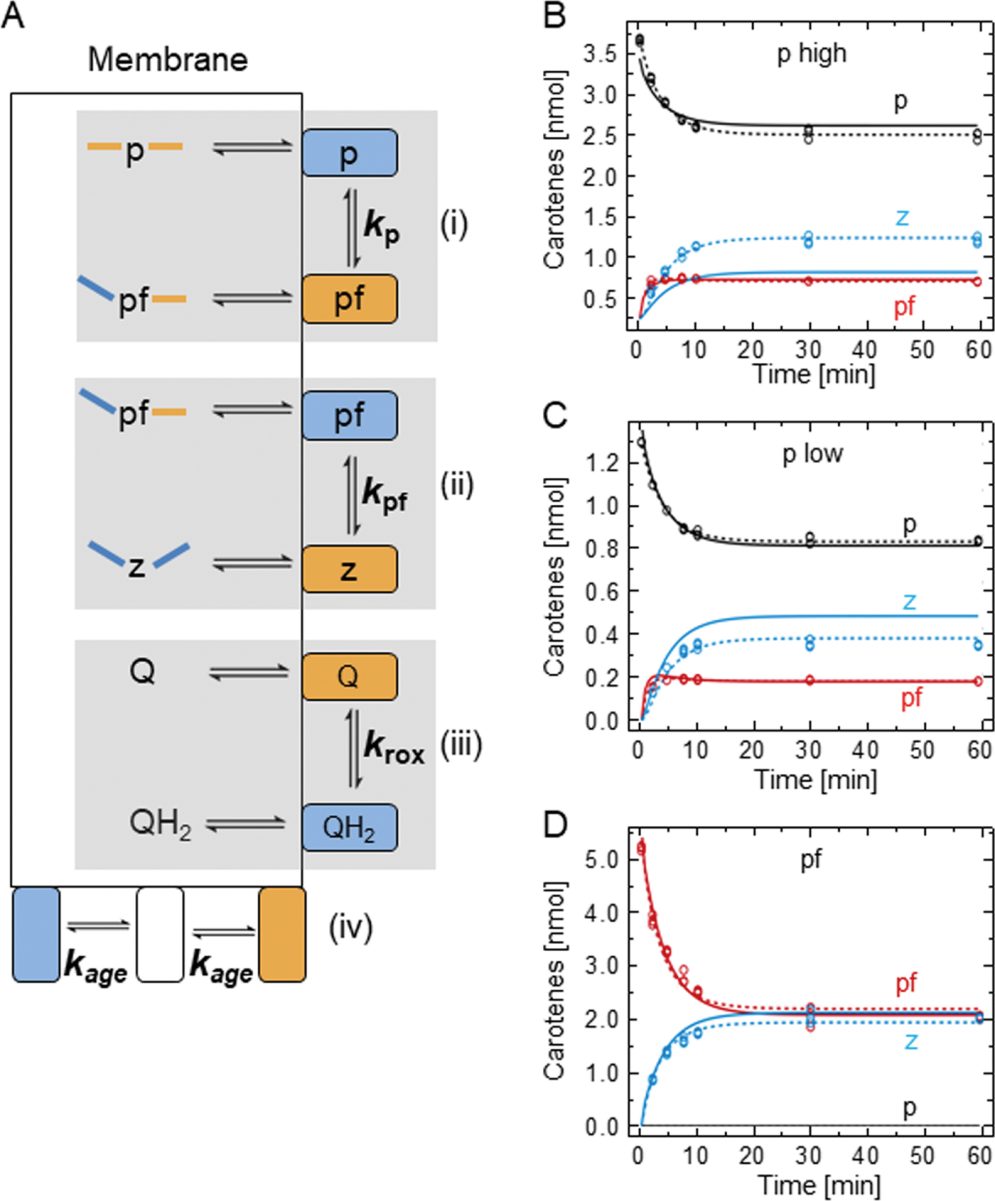
Kinetic scheme of the monomeric model and dynamic modeling of PDS reaction time courses. (A) Monomeric model. PDS monomeric subunits (orange and blue rectangles) within the homotetramer are assumed to work independently. Orange/blue color denotes reduced/oxidized half sides of phytoene (p), phytofluene (pf) and ζ-carotene (z) and the respective redox state of the PDS-bound FAD. The overall reaction comprises the three main processes phytoene desaturation (i), phytofluene desaturation (ii) and plastoquinone reduction (iii) with the rate constants *k*_p_, *k*_pf_ and *k*_rox_, respectively. Each rate constant encompasses the three equilibria represented by the reaction arrows associated to each of the three main processes which are highlighted by shadowed areas: association-dissociation of enzyme and substrate, desaturation-saturation of substrate and dissociation-association of enzyme and product. All hydrophobic carotene substrates and DPQ (Q) are soluble in the hydrophobic core of liposomal membranes. Progressive inactivation of PDS by denaturation (iv) is a process to be considered. (B-D) Reaction time courses of phytoene and phytofluene conversion by PDS. Reaction time courses were initiated [p] = 3.7 nmol (p high; B), [p] = 1.3 nmol (p low; C) and [pf] = 5.2 nmol (pf; D). The observables are given as data points (black, phytoene, p; red, phytofluene, pf; blue, ζ-carotene, pf), the model fit (obtained with model I; ODE 1–5) is represented by lines. The modeling was either based on simultaneous parameter estimation for all three reaction time courses (solid lines) or on simultaneous estimation of *k*_p_, *k*_rox_ and *k*_age_ and individual estimation of *k*_pf_ (dashed line). Measurements were carried out in triplicate.

To tailor the model complexity to the information content of the data, only the most relevant processes were included. Successive rounds of model reduction and reevaluation indicated the feasibility of condensing sub-processes into one rate constant as indicated by the grey shadowed areas in Fig 4A. It contains four rate constants for the main processes (i-iii) (Fig 4A) and (iv) enzyme inactivation. The latter was implemented by decreasing the amount of oxidized and reduced PDS over time with a rate constant k_age_ (see Eq 4 and 5). This “monomeric model” is represented by a set of five ordinary differential equations (ODEs) to model the time-dependent occurrence of p, pf, z and of oxidized and reduced PDS.

$$\frac{d}{dt}[p]=-k_{p}∙[p]∙[{FAD}_{ox}]$$ (1)

$$\frac{d}{dt}[pf]=-k_{pf}∙[pf]∙[{FAD}_{ox}]+k_{p}∙[p]∙[{FAD}_{ox}]$$ (2)

$$\frac{d}{dt}[z]=k_{pf}∙[pf]∙[{FAD}_{ox}]$$ (3)

$$\frac{d}{dt}[{FAD}_{ox}]=-k_{p}∙[p]∙[{FAD}_{ox}]-k_{pf}∙[pf]∙[{FAD}_{ox}]+k_{rox}∙[Q]∙{[FAD}_{red}]-k_{age}∙[{FAD}_{ox}]$$ (4)

$$\frac{d}{dt}[{FAD}_{red}]=k_{p}∙[p]∙[{FAD}_{ox}]+k_{pf}∙[pf]∙[{FAD}_{ox}]-k_{rox}∙[Q]∙{[FAD}_{red}]-k_{age}∙[{FAD}_{red}]$$ (5)

The observables are the HPLC-quantified amounts of p, pf and z in the reaction time courses. In addition, the initial amounts in the assay are: oxidized PDS monomers (FAD_ox, t0_; 0.18 nmol), of reduced PDS (FAD_red, t0_; 0 nmol), of membrane-soluble DPQ (Q_t0_; 9.63 nmol) as well as of the PDS products pf_t0_ and z_t0_ (0 nmol).

DPQ reduction with *k*_rox_ yields DPQH_2_ that could possibly be reoxidized non-enzymatically in the liposomal membranes of the assay. In order to investigate the role of the DPQ redox state, the two extreme scenarios, namely “no DPQH_2_ reoxidation” and “fast DPQH_2_ reoxidation”, were tested by modeling–the former corresponding to a maximally decreasing DPQ level during reaction time courses and the latter scenario corresponding to a constant DPQ level throughout reaction time courses. According to the model, no difference was found for the two scenarios regarding goodness of fit and rate constant values. In summary, the parameter for DPQH_2_ reoxidation is not identifiable, i.e. the model cannot distinguish between both scenarios. This is most likely because DPQ is present in large molar excess relative to the carotene substrates p and pf. However, additional experimental data at low concentrations of the electron acceptor DPQ supported rapid non-enzymatic reoxidation. For instance, 0.3 nmol DPQ in a standard assay resulted in 7.7 nmol of introduced double bonds. Based on the 2 e^-^ transfer involved in both carotene desaturation and DPQ reduction, one DPQ thus allows completing 26 carotene desaturation reactions. In conclusion, the amount of DPQ was held constant (Q_t0_ = 9.63 nmol) for the modeling of PDS reaction time courses.

The Eqs (1–5) were used to fit each reaction time course (p high, p low and pf) individually, i.e. by individual parameter estimation, yielding a good fit. However, fitting all three reaction time courses simultaneously, i.e. by simultaneous parameter estimation, reveals the imperfections of the monomeric model (solid lines in Fig 4B–4D). While phytofluene formation is generally well fitted (Fig 4B–4D) and so is ζ-carotene formation for “pf” (Fig 4D), it fails to adequately describe the formation of the latter for “p low” and “p high” (Fig 4A and 4B). Subsequent evaluation revealed that an individual estimation of *k*_pf_ for each single reaction time course was sufficient to fit the data, while all other parameter values could be estimated simultaneously (dotted line in Fig 4). The deduced rate constant values for the monomeric model, with the varying *k*_pf_ values for the three reaction time courses, are summarized in Table 1. The difference between *k*_pf_ in the “p low” and “p high” reaction time courses is insignificant, both being ca. 5 nmol^-1^ min^-1^. In contrast, *k*_pf_ for the “pf” reaction time course is as slow as 1.1 ± 0.1 nmol^-1^ min^-1^. Consequently, the conversion of pf produced from phytoene by PDS catalysis proceeds 5 x faster at the same concentration of reactants than the conversion of pf that was deposited in liposomes. The model thus requests two kinetically inequivalent phytofluene species for simultaneous fitting. This suggests that the desaturation of phytofluene might occur with different rates depending on whether it was experimentally provided as a freely diffusible substrate within membranes (as in “pf”) or whether it was “nascent” i.e. derived from phytoene desaturation by PDS catalysis (as in “p high” and “p low). The latter species gains access to PDS more readily.

**Table 1:** Parameter values for the monomeric and the substrate channeling model.

Monomeric model		Substrate channeling model	
Parameter	Value	Parameter	Value
***k*_p_**	0.54 ± 0.02 nmol^-1^ min^-1^	***k*_p_**	0.55 ± 0.02 nmol^-1^ min^-1^
***k*_pf_ (pf)**	1.14 ± 0.04 nmol^-1^ min^-1^	***k*_pf_**	1.15 ± 0.04 nmol^-1^ min^-1^
***k*_pf_ (p high)**	5.10 ± 0.24 nmol^-1^ min^-1^		
***k*_pf_ (p low)**	4.77 ± 0.22 nmol^-1^ min^-1^		
-	-	*k*_pf*_	5.44 ± 0.32 nmol^-1^ min^-1^
-	-	***k*_diff_**	0.02 ± 0.01 min^-1^
***k*_rox_**	5.76 (− 1.92 + 5.84) nmol^-1^ min^-1^	***k*_rox_**	5.40 (− 1.86 + 5.67) nmol^-1^min^-1^
***k*_age_**	0.22 ± 0.01 min^-1^	***k*_age_**	0.22 ± 0.01 min^-1^

**Fig 5:**
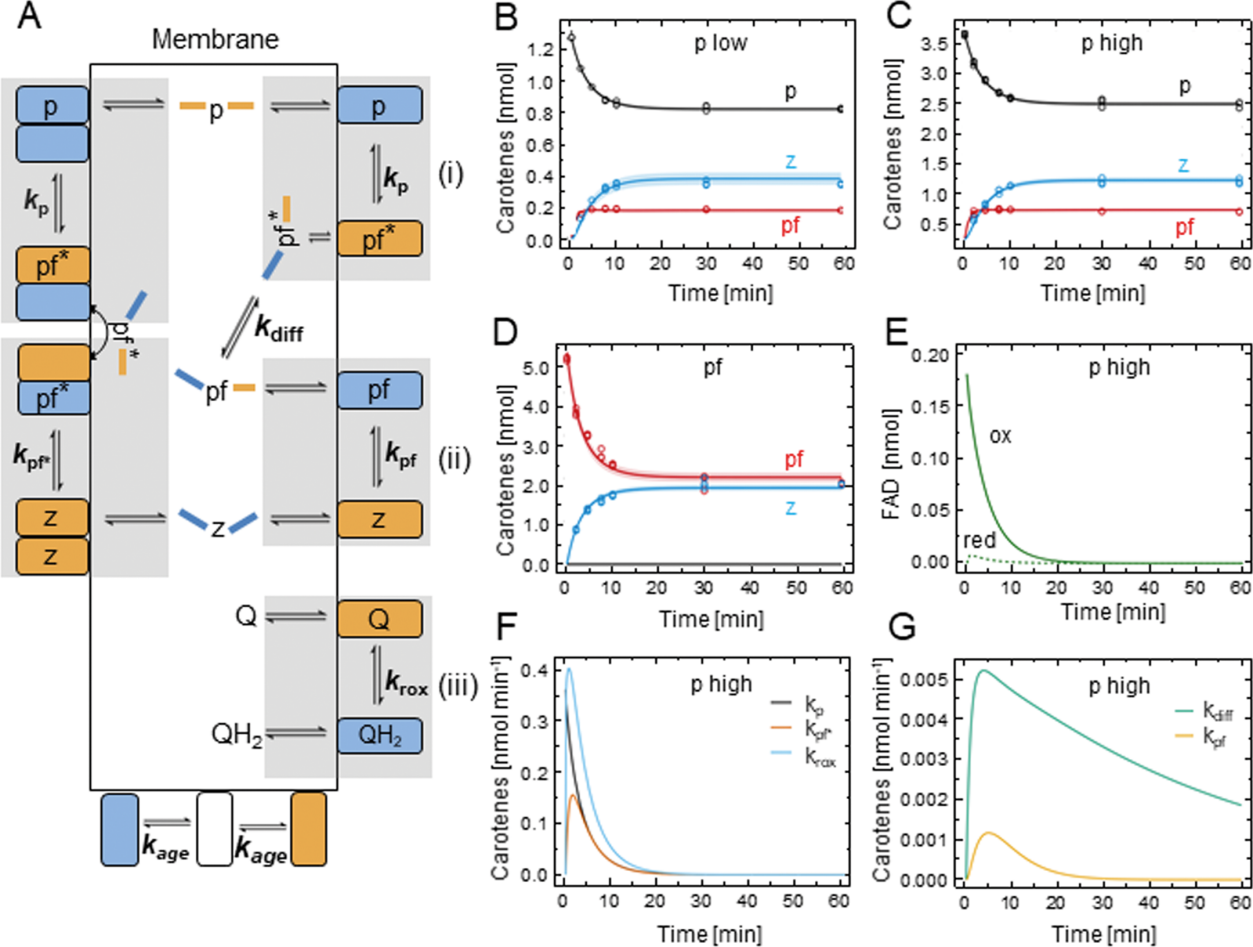
Kinetic scheme of the substrate channeling model and dynamic modeling of PDS reaction time courses. (A) Substrate channeling model, accounting for substrate channeling between PDS homotetramers. Symbols are as given in Fig 4A. Two species of phytofluene, i.e. phytofluene fates, coexist. Left; nascent phytofluene (pf*) that is produced from phytoene (p) can be restricted in its diffusion into the membrane residing in a microdomain in proximity to the PDS homotetramer, as indicated by the bent arrow. It can be channeled into a second PDS subunit of the homotetramer containing FAD_ox_, allowing rapid conversion to ζ-carotene (z) with the rate constant *k*_pf*_. Right; pf* can alternatively diffuse into PDS-distant membrane areas with rate constant *k*_diff_, this defining the species pf. From there it can be taken up by another monomeric PDS subunit and be converted into ζ-carotene (z) with rate constant *k*_pf_. Rate constant *k*_age_ represents enzyme inactivation which refers to both the reduced and oxidized enzyme states. (B-G) Dynamic modeling of reaction time courses of phytoene and phytofluene conversion by PDS. Reaction time courses were conducted with 1.3 nmol phytoene (p low; B), and 3.7 nmol phytoene (p high; C). In addition, liposomes containing 5.2 nmol phytofluene were used (pf; D). The observables are given as data points (black, phytoene, p; red, phytofluene, pf; blue, ζ-carotene, z). The model fit, represented by lines, is based on Eqs 1 and 6–10 with simultaneous parameter estimation for all three reaction time courses. Shadowed areas indicate one standard deviation as estimated by the error model (see Methods). Measurements were carried out in triplicate. (E) Prediction of the amount of oxidized, active PDS (ox) and reduced PDS (red) over time, indicating a rapid decrease in oxidized and reduced PDS levels due to enzyme inactivation. (F,G) Deduced carotene fluxes through the different sub-processes labeled with their rate constants (see Fig 4). Note the different scaling in F and G. Flux predictions are based on the phytoene conversion reaction time course “p high” (C).

Parameter values derived from the monomeric model (Fig 4A) and substrate channeling model (Fig 5A) are given. They are based on the reaction time courses “pf” using liposomes containing 5.2 nmol phytofluene per assay as well as “p high” and “p low” in which the phytoene conversion in liposomes containing 3.7 nmol phytoene (p high) and 1.3 nmol phytoene (p low) was measured. Estimated parameter values are given ± 1 ơ confidence intervals. For the monomeric model, simultaneous parameter estimation for all reaction time courses was applied to *k*_rox_ and *k*_age_, assuming that FAD reoxidation and enzyme inactivation are independent of the carotene substrate present (p or pf), and to *k*_p_. Individual parameter estimation for every reaction time course was applied to *k*_pf_. For the substrate channeling model, simultaneous parameter estimation across all reaction time courses was applied (Fig 5).

Guided by these findings and by homotetrameric assembly of PDS at membrane surfaces [6, 16], the PDS reaction scheme was refined (Fig 5A). Starting from phytoene as the substrate, a phytofluene species pf* was introduced that is characterized by limited diffusion within the lipid bilayer. It can be channeled between PDS subunits to be more rapidly converted into ζ-carotene with rate constant *k*_pf*_ (Fig 5A, left). This species might reside in a membrane domain that is organized by the bound tetramer. In addition, non-channeled phytofluene desaturation takes place relying on phytofluene pf* that “escapes” from this domain to diffuse freely within the plane of the lipid bilayer (Fig 5A, right). Release of nascent phytofluene pf* into the membrane occurs with rate constant *k*_diff_. The released phytofluene, now termed pf, defines a species of the intermediate that is more slowly converted into ζ-carotene than pf*, with pf being converted with rate constant *k*_pf_. This diffusing species pf would be equivalent to the phytofluene experimentally provided within liposomes as substrate. It is to be understood that phytofluene detected during PDS reaction time courses comprises both pf and pf*. The resulting mathematical model (substrate channeling model) combines both fates of phytofluene and is represented by the following ODEs in addition to Eq (1):

$$\frac{d}{dt}[{pf}^{*}]=-k_{{pf}^{*}}∙[{pf}^{*}]∙[{FAD}_{ox}]-k_{diff}∙[{pf}^{*}]+k_{p}∙[p]∙[{FAD}_{ox}]$$ (6)

$$\frac{d}{dt}[pf]=-k_{pf}∙[pf]∙[{FAD}_{ox}]+k_{diff}∙[{pf}^{*}]$$ (7)

$$\frac{d}{dt}[z]=k_{pf}∙[pf]∙[{FAD}_{ox}]+k_{{pf}^{*}}∙[{pf}^{*}]∙[{FAD}_{ox}]$$ (8)

$$\frac{d}{dt}[{FAD}_{ox}]=-k_{p}∙[p]∙[{FAD}_{ox}]-k_{pf}∙[pf]∙[{FAD}_{ox}]-k_{{pf}^{*}}∙[{pf}^{*}]∙[{FAD}_{ox}]+k_{rox}∙[DPQ]∙{[FAD}_{red}]-k_{age}∙[{FAD}_{ox}]$$ (9)

$$\frac{d}{dt}[{FAD}_{red}]=k_{p}∙[p]∙[{FAD}_{ox}]+k_{pf}∙[pf]∙[{FAD}_{ox}]+k_{{pf}^{*}}∙[{pf}^{*}]∙[{FAD}_{ox}]-k_{rox}∙[DPQ]∙[{FAD}_{red}]-k_{age}∙[{FAD}_{red}]$$ (10)

The substrate channeling model shows to fit of all three reaction time courses using a single set of rate constants (Fig 5B–5D). The parameter values are provided in Table 1 and the corresponding parameter likelihood profiles are given in S3 Fig, demonstrating that all parameters are well defined. The data show that the conversion of lipid-diffusible phytoene p (k_p_ ≈ 0.55 nmol^-1^ min^-1^) is slower than the conversion of lipid-diffusible phytofluene pf (k_pf_ ≈ 1.15 nmol^-1^ min^-1^). However, both substrates, experimentally provided in liposomal membranes, are converted at slower rates than the “nascent” phytofluene pf* species (k_pf*_ ≈ 5.44 nmol^-1^ min^-1^). In an interpretation, the restricted diffusion of the latter would increase its local concentration, allowing another subunit of the same PDS homotetramer to accelerate phytofluene conversion by a factor of 5. Notably, the reoxidation of FAD_red_ in PDS is comparatively fast with *k*_rox_ of 5.40 nmol^-1^ min^-1^ and up to 11.17 nmol^-1^ min^-1^ within one standard deviation.

Only a very small proportion of PDS is in its reduced state during reaction time courses (Fig 5E) as witnessed by the high reoxidation flux through *k*_rox_ keeping up with PDS reduction by carotene desaturation (compare with fluxes through *k*_p_ and *k*_pf*_; Fig 5F). This suggests that PDS reoxidation is not rate-limiting. Regarding PDS inactivation *in vitro*, a rapid decrease of both oxidized and reduced PDS is suggested by *k*_age_ of 0.22 min^-1^, resulting in a half life of approximately 4 min (Fig 5E). The rate constant *k*_diff_ (≈ 0.02 min^-1^), representing the release of nascent pf* from the microdomain into the membrane as freely diffusing pf, suggests that 2% of pf* leave the microdomain each minute. This favors channeled conversion of pf* into ζ-carotene with *k*_pf*_. Accordingly, the calculated carotene fluxes through all desaturation processes (see Fig 5F and 5G) show that the pf* flux into ζ-carotene through k_pf*_ exceeds by far the phytofluene fluxes through *k*_diff_ and *k*_pf_. Thus, the channeling of the intermediate pf* facilitates and accelerates end product formation and represents a necessary process in the model to describe PDS reaction time courses.

Taken together, the substrate channeling model is consistent with deductions made from the PDS crystal structure (see Introduction) by corroborating the relevance of oligomeric assemblies of PDS at the surface of liposomes. The catalysis by PDS relies on a metabolite channel to favor end product over intermediate formation.

### Simulation of substrate concentration dependencies

PDS catalyzes a bi-substrate reaction involving a carotene, phytoene (p) or phytofluene (pf), and a benzoquinone (DPQ). To investigate the concentration-dependent behavior of PDS, pseudo-first order conditions were attained by using the invariable substrate at saturating concentrations. We furthermore stress-tested the validity of the mathematical model by investigating whether also concentration dependencies could adequately be simulated. For this, phytofluene and ζ-carotene formation was simulated based on the rate constants and the initial amounts of substrates and enzyme.

**Fig 6:**
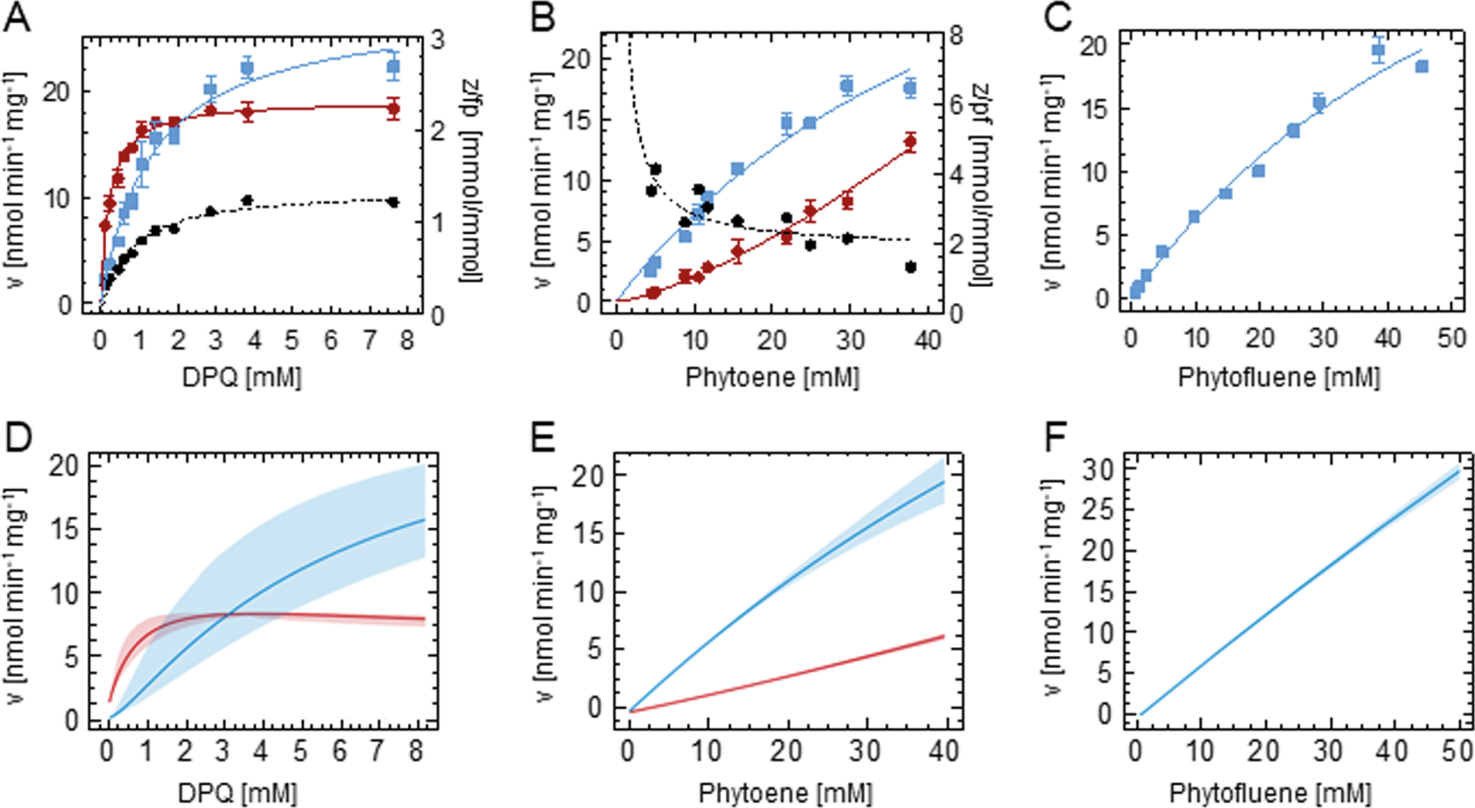
Data and model predictions on concentration-dependent PDS reaction rates. Measured (A-C) and simulated (D-E) concentration dependency of the PDS reaction rates. Dependency on (A) DPQ determined at [p] = 40 mM (≈ 1 x K_M_), (B) phytoene measured at [DPQ] = 19.25 mM (≈ 15 x K_M_) and (C) phytofluene measured at [DPQ] = 19.25 mM. Data represent triplicates ± SEM. Phytofluene and ζ-carotene formation in A–C were fitted with the MM equation (see Methods; solid lines; goodness of fit for ζ-carotene formation: A, R^2^ = 0.98; B, R^2^ = 0.97; C, R^2^ = 0.98) except phytofluene formation in B that was fitted with a spline. The ζ-carotene:phytofluene ratios in A and B are given as dotted lines and plotted to the right y-axis. Date are given as squares the solid lines represent the fit (A-C) or model prediction (D-F). Red color denotes ζ-carotene; blue represents phytofluene. Shadowed areas in D—F represent one standard deviation.

DPQ dependency was examined at the maximally attainable phytoene concentration of 40 mM; higher concentrations led to liposome precipitation. The formation of the end product ζ-carotene can be fitted with the Michaelis-Menten (MM) equation (Fig 6A). Since phytofluene as the intermediate is in steady state and is subjected to two different fates (see above), it is not astonishing that its formation does not show MM conformity. Consequently, product:intermediate ratios vary substantially (dotted line in Fig 6A), with increasing DPQ concentrations favoring end product formation. Simulation of the DPQ dependency by use of the mathematical model revealed the same trend (compare Fig 6A and 6D). While the observed and estimated apparent V_max_ values are also very similar (Table 2) there is a ca. 4-fold difference in K_M_. The dependency of the PDS reaction rate on the concentration of phytoene and phytofluene was examined at a saturating DPQ concentration ([DPQ] = 19.25 mM ≈ 15 x K_M_; Fig 6B and 6C). Both carotene substrate concentrations cannot be increased to saturation for reasons of liposome integrity (see above). Fitting ζ-carotene formation from phytoene with the MM equation (Fig 6B) allows determining apparent phytoene K_M_ and V_max_ values that are in a reasonable agreement with those obtained from simulation (Table 2). Again, the formation of the intermediate phytofluene showed no MM conformity. Notably, no sigmoidality–a hallmark of cooperative substrate binding in oligomeric enzymes–was observed for ζ-carotene formation.

**Table 2:** Observed and estimated apparent K_M_ and V_max_ values for PDS substrates.

Substrate	Enzyme	K_M_ exp.	K_M_ sim.	V_max_ exp.	V_max_ sim.
		[mM]	[mM]	nmol min^-1^ mg^-1^]	nmol min^-1^ mg^-1^]
**DPQ**	wild type	1.3 ± 0.2	6.2 ± 2.5	28.1 ± 1.4	26.2 ± 0.8
	Arg300Ser	0.4 ± 0.1*	-	1.2 ± 0.1*	-
**Phytoene**	wild type	53.9 ± 18.1	71 (–27 +160)	46.3 ± 10.7	51 (–18 +43)
	Arg300Ser	4.5 ± 2.6	-	1.0 ± 0. 2	-
**Phytofluene**	wild type	66.8 ± 20.7	126 (–40 +120)	48.4 ± 10.3	195 (–67 +187)
	Arg300Ser	-	-	-	-

Apparent K_M_ and V_max_ values were estimated based on ζ-carotene formation for the observed and estimated concentration dependencies of PDS (Fig 6). The mean values ± SD are given. For the experimental data, the MM equation (see Methods) was used. Simulated values were obtained from the mathematical model.

* indicates that [p] = 10 mM was used for Arg300Ser, in contrast to [p] = 40 mM for wild type. exp., experimental; sim., simulated.

As observed with DPQ, substrate concentration affects the product:intermediate ratio due to the different kinetics of phytofluene and ζ-carotene formation. Increasing phytoene concentrations favor phytofluene release, with the z:pf ratio decreasing from ca. 4:1 to 1:1 (Fig 6B, dotted line). These relations are well reflected in the simulation (compare Fig 6B and 6E). This is compatible with the notion of PDS being a bifunctional phytoene-phytofluene desaturase with both carotenes competing for enzyme binding. In an interpretation of the substrate channeling model, these findings suggest that low carotenoid fluxes through PDS and high DPQ concentrations favor end product formation, while the opposite favors intermediate release. With phytofluene as initial substrate, the rate of ζ-carotene formation can be fitted satisfactorily using the MM equation (Fig 6C), allowing an estimation of apparent K_M_ and V_max_ values that differ from those derived from simulation (compare Fig 6C and 6F; Table 2).

In summary and in support of the validity of the model, the concentration-dependent correspondence of rates of intermediate and end product formation are well reflected across all simulations. However, it overestimates MM parameters, used here for comparisons, by factors of 1.1 to 4.1 (Table 2). This systematic error is likely due to continuous structural alterations caused by the incorporation of increasing concentrations of the poly-*cis*-configured long-chain hydrocarbon substrates into liposomes [35] that can interfere with PDS activity. However, the model has been established with reaction time courses in the lower range of substrate concentrations and cannot consider this structural circumstance upon extrapolation. Moreover, the production of enzyme and / or liposomes–this cannot be distinguished because of their mutual dependency in the biphasic system used–with identical specific activities from batch to batch showed to be notoriously difficult. The one used in the reaction time course experiments to develop the model was different from the one used in concentration dependency experiments. This fact can as well contribute to the quantitative deviations from the model, while qualitative similarities are being maintained.

### The norflurazon mode of inhibition and effects of active site mutations are compatible with ordered ping-pong bi-bi and “flavin only” mechanisms

**Fig 7:**
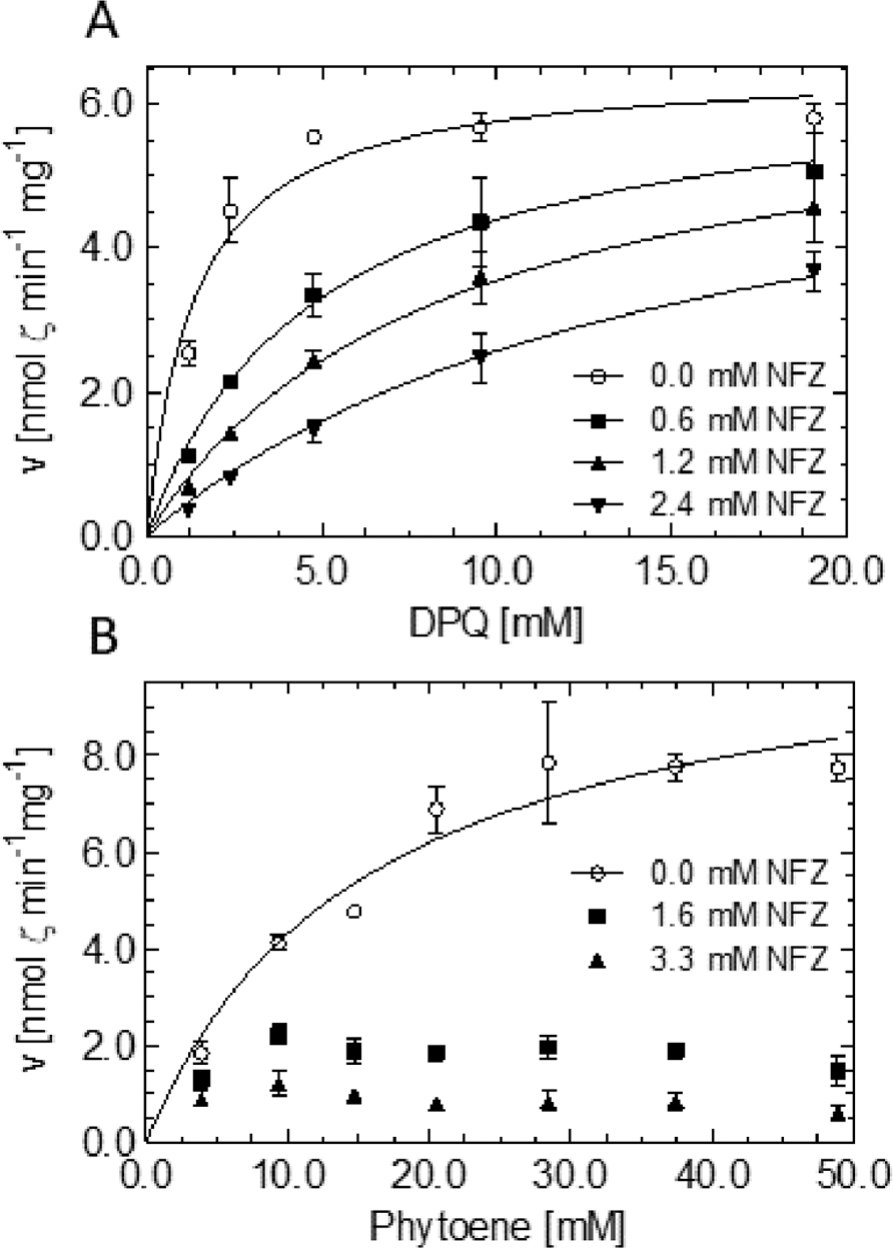
DPQ and phytoene concentration dependencies of PDS inhibition by NFZ. PDS inhibition was investigated at the indicated increasing concentrations of the inhibitor NFZ and of the substrates (A), DPQ and (B), phytoene. Data represent triplicates ± SEM and were fitted with the equation for competitive inhibition (A; R^2^ = 0.99) and the Michaelis-Menten equation (B; 0.95) using the GraphPad Prism 5 software. Data obtained in the presence of NFZ in B were not fitted due to poor goodness of fit with the equations for competitive, non-competitive and uncompetitive inhibition (for equations, see Methods). All other assay parameters were as defined (for standard conditions, see Methods).

The crystal structure of OsPDS-His_6_ [16] implies that all substrates occupy the same cavity in sequential order to access the FAD-containing active center. NFZ occupies the DPQ binding site within this cavity. Consequently, NFZ should be competing with DPQ. Moreover, NFZ might as well interfere with the binding of the carotene substrate phytoene. The data of Fig 7A show that NFZ behaves competitively with DPQ inhibiting with a K_i_ = 0.23 ± 0.03 mM supporting previous evidence [6]. Other *meta*-trifluoromethylphenyl–containing PDS inhibitors such as fluridone and diflufenican, also thought to occupy the plastoquinone binding site [36], behaved similarly. In contrast, at increasing phytoene concentrations, V_max_ could not be attained in the presence of NFZ (Fig 7B). This suggests that the inhibition observed is not competitive with phytoene. However, neither non-competitive nor uncompetitive models were able to adequately describe the observed inhibition kinetics. A non-competitive inhibition would be supported by the fact that the PDS-NFZ crystal structure represents an enzyme-inhibitor complex formed in the absence of substrate [16].

In summary, NFZ competes with DPQ but does not compete with phytoene, although all three bind to the same cavity. This supports the proposed ordered ping-pong bi-bi mechanism, i.e. a sequential binding of phytoene to the oxidized and DPQ (or its competitor NFZ) to the reduced state of the enzyme. It is conceivable that the redox state of FAD may act as a switch triggering conformational changes between folds that preferentially bind the carotene (FAD_ox_) or PQ (FAD_red_).

PDS mutations conferring resistance to NFZ have been identified in cyanobacteria [17, 18], algae [37–39] and in one aquatic plant [19]. The reported mutations pertain to five highly conserved amino acids, Phe162, Arg300, Leu421, Val505 and Leu538 (numbering according to the *O*. *sativa* enzyme; S4 Fig). According to the structure of the PDS-NFZ complex [16], these residues are localized in the environment of the active center (S5 Fig). Among these residues, Arg300 coordinates NFZ via hydrogen bonding and is assumed to participate in binding and possibly promoting the reactivity of the terminal electron acceptor plastoquinone [16]. Due to its position in the active site, Arg300 represents the only residue that might be mechanistically involved in initiating carotene desaturation by acid-base catalysis (S5 Fig; [16], as suggested for the bacterial carotene desaturase CRTI [7]. However, Arias et al. [19] reported that PDS maintains substantial activity upon substitution of the arginine corresponding to Arg300 in *O*. *sativa* by virtually any other proteinogenous amino acid. This is inconciliable with a decisive role in catalysis.

In order to investigate the role of Arg300 for the *O*. *sativa* enzyme, the mutations Arg300Ser and Arg300Thr were introduced. Most importantly, both of the purified PDS versions retained ζ-carotene forming activity under standard conditions, albeit at only 15% and 6% of the wild type enzyme, respectively. This defines Arg300 as not being catalytically essential and supports the “flavin only” mechanism in which a CH-CH single bond reacts spontaneously with the isoalloxazine [16]. However, the reduced activity might indicate ancillary functions such as polarizing the carotene π electron system, thereby facilitating desaturation of the adjacent C11-C12 site [16]. Retaining substantial activity, Arg300Ser was chosen for further characterization. Incorporation of FAD into the enzyme (see Methods) was not affected, with both wild type PDS and Arg300Ser being ca. 70% in the holo form. There was also no difference in membrane association (S6A Fig) and GPC analysis revealed that the mutation did not affect the oligomeric assembly and solubility of the protein either (S6B Fig). Therefore, Arg300Ser PDS most likely maintains a native overall fold.

**Fig 8:**
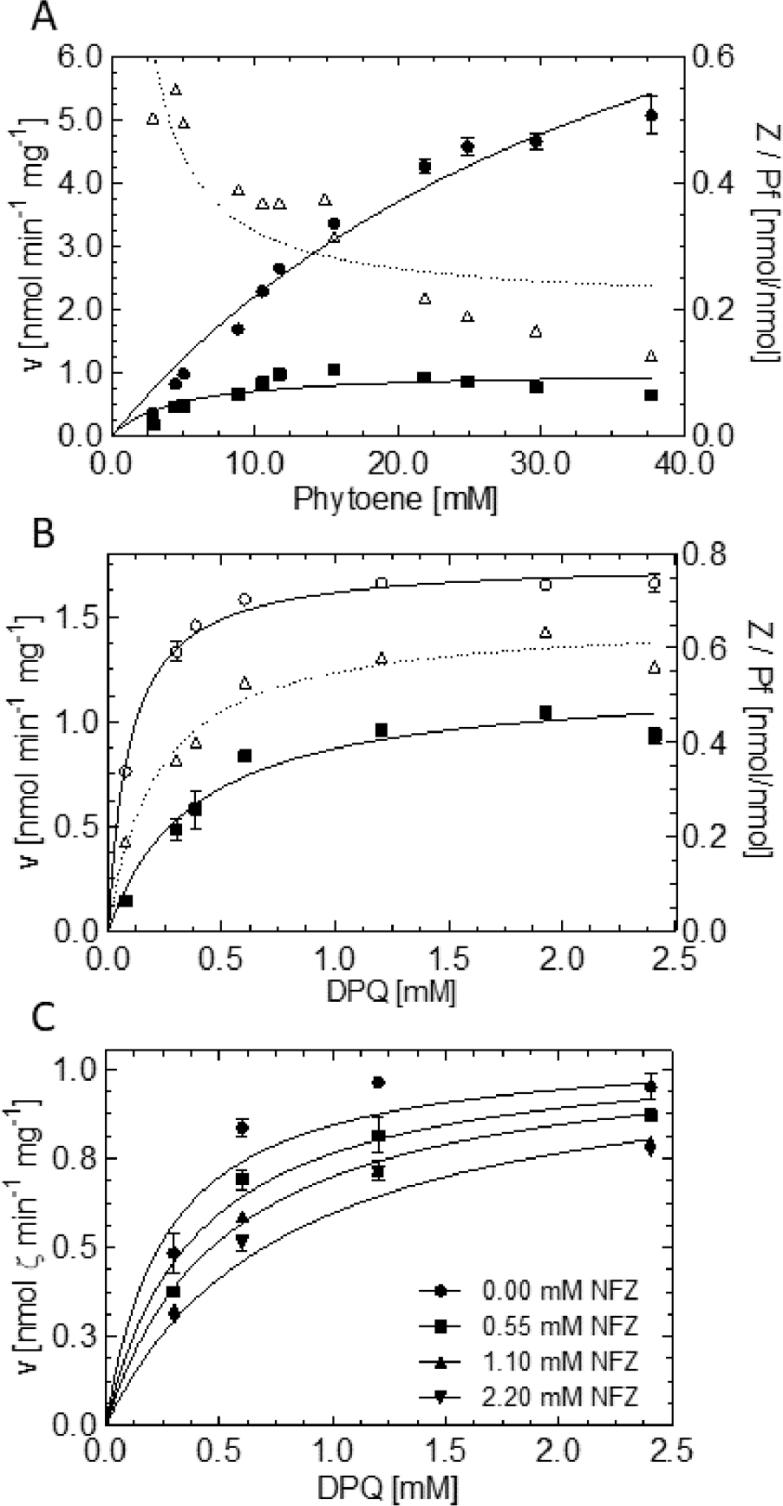
Kinetic characterization of Arg300Ser PDS. Dependence of Arg300Ser PDS reaction rates on phytoene (A) and DPQ (B). (C) Inhibition kinetics of Arg300Ser PDS in a matrix of varying DPQ and NFZ concentration. Data points represent the mean of duplicates ± SEM. In A and B: ■, ζ-carotene; ○, phytofluene; Δ, ζ-carotene:phytofluene ratio. Data in A (R^2^ = 0.58) and B (R^2^ = 0.95) were fitted with the Michaelis-Menten equation and the equation for competitive inhibition was applied in C (R^2^ = 0.92) using the GraphPad Prism 5 software (for equations, see Methods). The product:intermediate ratios in A and B (dotted lines; plotted to the right Y-axis) was fitted using a spline. Assays were carried out under standard conditions and incubated for 15 min (see Methods).

Based on the role of Arg300 in NFZ and (presumably) DPQ binding by H-bridge formation [16], the binding affinity for both ligands might be lowered. The substantially shorter side chain of Ser renders stable hydrogen bonds improbable. This substitution might confer NFZ resistance and concomitantly diminishes DPQ binding and consequently, FAD_red_ reoxidation. This might cause the impeded desaturation activity. In line with these expectations, the mutated enzyme revealed a ca. 5-fold increased resistance to NFZ with a K_i_ of 1.11 ± 0.36 mM (Fig 8C), compared to a K_i_ of 0.23 ± 0.03 with wild type PDS. However, in contrast to expectation, the apparent K_M_ for DPQ was decreased by factor of ca. 3.5 (Table 2). When interpreted in terms of ligand affinities, the increased NFZ K_i_ accompanied by the decreased DPQ K_M_ is not fully compatible with the notion of a simple analogy of NFZ and DPQ binding [16]. On the other hand, the removal of the charged residue increases lipophilicity of the active site. This might favor the binding of the very lipophilic DPQ and of carotene substrates over the less lipophilic NFZ. In line with this, the K_M_ for phytoene, occupying the same cavity, is likewise lowered (Fig 8A). The increased affinity for phytoene might be accompanied by an equivalently increased affinity for phytofluene and ζ-carotene. This might hinder carotene product release, diminish plastoquinone binding for FAD_red_ reoxidation and consequently, catalytic activity. This is mirrored by the decreased V_max_ for both substrates (Table 2). However, it also needs to be noted that the removal of a charge from an active center represents a major change possibly affecting longer-range conformational changes that can exert multiple effects. The predominance of phytofluene release by the Arg300Ser mutation (comp. Fig 6A and 6B with Fig 8A and 8B, see dotted lines representing product/intermediate ratios) might also point towards impaired substrate channeling. However, mathematical modeling did not allow distinguishing the responsible sub-processes.

Additional mutations, Leu538Phe, Leu538Arg and Phe162Val were introduced by site-directed mutagenesis, all reported to confer NFZ resistance. The mutant proteins were purified and showed to remain flavinylated. However, all of these variants showed < 5% of the wild type activity and could not be kinetically characterized. The complementation of these PDS variants in *E*. *coli*, engineered to produce 15-*cis*-phytoene, resulted in low levels of ζ-carotene formation which may be sufficient to observe the reported NFZ resistance in cyanobacteria [17–19, 26] and even plants. Thus, the gain in NFZ resistance trades-in lowered catalytic effectiveness, which might be compensated by transcriptional or other regulatory mechanisms in the parent biological backgrounds.

### The central 15-cis configuration of phytoene mediates the regio-specificity in catalysis

PDS catalyzes the introduction of double bonds exclusively at C11-C12 and C11’-C12’ of phytoene (Fig 1). Since neither PDS co-crystallization nor crystal soaking with its lipophilic substrate were successful, it remains elusive how the relative positioning of the C11-C12 carbon bond and the redox-reactive flavin moiety is achieved to attain the high regio-specificity observed. The length of the substrate cavity of approximately 43 Å suggests that phytoene is completely inserted in an extended conformation [16]. Correct substrate positioning might depend on substrate molecule length with some polar residues at the back end of the cavity acting as a restrictor for its insertion. Alternatively, the observed kink in the cavity might act as restrictor, corresponding to the position where the central 15-*cis* double bond of phytoene is arrested.

**Fig 9:**
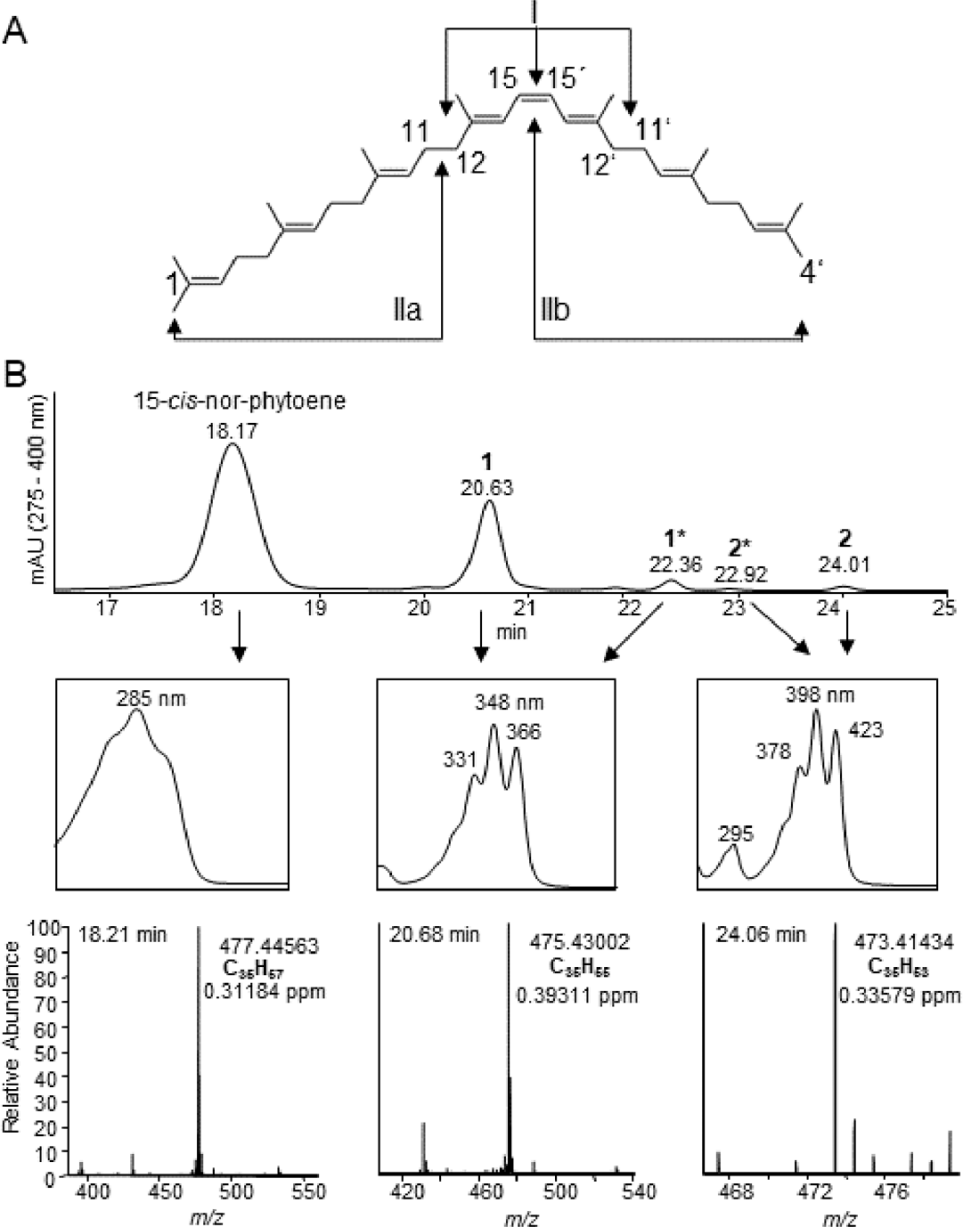
LC-MS analysis of PDS desaturation products produced from asymmetric (C_35_) 15-*cis*-nor-phytoene. (A) Structure of 15-*cis*-1',2',3',16',17'-penta-nor-phytoene (15-*cis*-nor-phytoene). The desaturation sites C11-C12 and C11’-C12’ and the central C15-C15’ double bond are marked. The carbon bonds located above the redox-reactive isoalloxazine are indicated by arrows if substrate positioning is mediated by the central 15-*cis*-configured triene (I) or substrate cavity back end (II). See text for details. (B) Identification of PDS desaturation products by LC-MS analysis. Carotenes were detected photometrically in the 275–400 nm range (top panel). The UV/VIS spectra of 15-*cis*-nor-phytoene and the desaturation products are shown (central panel). The bottom panel shows the corresponding MS^1^ spectra with the exact masses of the quasi-molecular ions [M+H]^+^, the derived sum formula and the mass deviation.

To elucidate the mechanistic aspect that determines regio-specificity, a C_5_-truncated variant of 15-*cis*-phytoene (15-*cis*-1',2',3',16',17'-penta-nor-phytoene; hereafter 15-*cis*-nor-phytoene; Fig 9A) was used as a substrate. Assuming the 15-*cis*-configuration as the decisive reference point, PDS would maintain specificity for C11-C12 and C11’-C12’ with 15-*cis*-nor-phytoene (Fig 9A, scenario I). The substrate would be desaturated twice, yielding an end product with a chromophore identical to that of 9,15,9’-tri-*cis*-ζ-carotene. However, if substrate length and the cavity back end are crucial for regio-specificity the reaction is expected to be disturbed when the truncated substrate half side is introduced first (Fig 9A, scenario IIb). In this case, the C11-C12 single bond of 15-*cis*-nor-phytoene would slip beyond the redox-active flavin moiety and instead, the central triene with C15-C15’ would occupy this position. Consequently, no carotene desaturation could occur. Upon introduction with the intact substrate half side first, regio-specificity for C11-C12 would be maintained and carotene desaturation can occur (Fig 9A, scenario IIa). Thus, the desaturation product of 15-*cis*-nor-phytoene would only be desaturated once and possess a pentaene with a phytofluene-like spectrum (Fig 9A).

15-*cis*-nor-phytoene was in fact converted by OsPDS-His_6_ and the desaturation products formed under standard conditions were characterized by LC-MS. The substrate 15-*cis*-nor-phytoene (Fig 9B) resembled 15-*cis*-phytoene regarding its UV/VIS spectrum and its [M+H]^+^ had the expected molecular mass corresponding to C_35_H_57_. In fact, two desaturation products were detected (Fig 9B). The [M+H]^+^ of the main product 1 is consistent with the loss of two hydrogens (C_35_H_55_) and the corresponding UV/VIS spectrum is similar to the one of 9,15-di-*cis*-phytofluene. This was accompanied by certain amounts of product 1* with identical UV/VIS spectra and molecular masses, most likely a different *cis* isomer of 1. The second product 2, also consisting of two isomers with identical properties, reveals a [M+H]^+^ that is consistent with the loss of another two hydrogens (C_35_H_53_) and showed a spectrum strongly resembling 9,15,9’-tri-*cis*-ζ-carotene. Taken together, these results indicate that regio-specificity for C11-C12 and C11’-C12’ is maintained with the truncated phytoene, i.e. that the central 15-*cis-*configured triene acts as a reference point for substrate positioning in the kinked substrate cavity.

To investigate whether the substrate cavity back end co-determinates regio-specificity, mutations were introduced at this site. It is characterized by polar amino acids such as the conserved Tyr506 and Thr508 that coordinate water molecules [16]. Replacing them by Phe and Val, respectively, generates a more hydrophobic cavity end and prevents water coordination. This might enable deeper substrate introduction and altered regio-specificity. However, using phytoene as a substrate, the mutant enzyme formed 9,15-di-*cis*-phytofluene and 9,15,9’-tri-*cis*-ζ-carotene as the sole products. This can be interpreted in support of a decisive role of the 15-*cis*-configured central triene rather than of the cavity back end, although the mutant enzyme exhibited low activity (ca. 5% ζ-carotene of wild type OsPDS-His_6_).

### Possible regulatory significance of intermediate leakage

By its kinetic properties PDS forms a leaky metabolite channel at membrane surfaces that is dependent on a microdomain orchestrated by the tetrameric assembly creating a “sink” for phytofluene. The imperfection of the system might be relevant. From the data presented, phytofluene appears as the candidate for a released signaler of system overflow caused by too high phytoene concentrations and/or too low quinone availability (Fig 6A and 6B). The inverse might signal too low biosynthetic activity. This suggestion is raised here in the light of recent publications indicating a signaling function stemming from *cis*-configured desaturation intermediates [40, 41]. However, the jury is still out on this issue, in the absence of knowledge on *cis*-phytofluene metabolizing steps, expectedly involving cleavage [42]. Moreover, the perception of this intricate desaturation system may change over time, when more structural and kinetic information becomes available on the enzymes downstream of PDS, i.e. ZISO, ZDS, CRTISO and LCY. Especially the question, whether all of these enzymes form defined supramolecular complexes at membrane surfaces remains to be substantiated.

## Supporting information

S1 Fig Postulated kinetic events during the ordered ping-pong bi-bi mechanism of PDS. The PDS monomer has one long substrate channel with oxidized FAD near the bottom end. Phytoene is symmetric as indicated by the two arms (blue color and orange colors denote oxidized and reduced states, respectively). The carotene enters with one oxidized (saturated) end and is desaturated, thereby reducing FAD (ping). The resulting phytofluene retains one oxidized end and is expelled into the lipid phase. The channel can now be occupied by plastoquinone to oxidize FAD_red_ (pong) and to reconstitute the oxidized enzyme for a new round of catalysis. Because of this temporally separated succession of events, the two redox reactions are thought to be thermodynamically independent. The intermediate phytofluene, still possessing one half side being identical to that of phytoene, can as well be a PDS substrate by entering the substrate cavity with the saturated end. Increasing phytofluene amounts can therefore compete with phytoene for desaturation.
(DOCX)

S2 Fig Conversion of 9,9’-di-*cis*-ζ-carotene by daffodil chromoplasts. 9,15,9’-tri-*cis*-ζ-carotene was purified from OsPDS-His_6_ assays (see Methods), photoisomerized to 9,9’-di-*cis*-ζ-carotene in day light and used as substrate with chromoplasts as described elsewhere [24]. The upper HPLC trace (HPLC system 4) represents a control assay incubated in the absence of the substrate showing background levels of prolycopene (1), proneurosporene (2) and of ζ-carotene isomers (3). The increased presence of (1) and (2) indicate the stereospecific identity of the 9,9’-di-*cis*-ζ-carotene added. The amount of ζ- (4) and β-carotene (5) present cannot change in aerobic assays [24] and therefore serve as an internal reference. The UV/VIS spectra of the substrate and the products are given as insets.
(DOCX)

S3 Fig Parameter likelihood profiles for the estimated dynamic parameters deduced from the substrate channeling model. The profile likelihood, χ^2^, is plotted over a range of parameter values around the estimated optimal value marked by a dot. As reference, the 68% / 90% / 95% confidence level (CL) thresholds corresponding to χ^2^ = 1 / 2.71 / 3.84 are given as horizontal lines.
(DOCX)

S4 Fig Section of the protein alignment for PDS from *Oryza sativa* and cyanobacteria, algae and plants with reported mutations conferring NFZ resistance. The following residues are highlighted: 1, Phe_162_; 2, Arg_300_; 3, Tyr_506_; 4, Thr_508_ 5, Leu_538_. Global sequence alignment was carried out with the Blosum62 matrix. Identical residues are green, similar residues greenish or yellow. Position numbering refers to the immature protein from *O*. *sativa* (A2XDA1.2) including its N-terminal 87 amino acid transit peptide. Organisms and accession numbers (from top to bottom): *Oryza sativa*, A2XDA1.2; *Arabidopsis thaliana*, Q07356.1; *Chlorella zofingiensis*, ABR20878.1; *Hydrilla verticillata*, AAT76434.1; *Synechococcus elongatus* PCC 7942, CAA39004.1; *Synechocystis sp*. PCC6803, CAA44452.1.
(DOCX)

S5 Fig Substrate cavity of OsPDS-His_6_ containing the redox cofactor FAD. The inner surface of the PDS substrate cavity is depicted. The substrate cavity entry in the membrane binding domain is indicated by an arrow. The redox cofactor FAD is given as sticks representation in orange. Conserved residues whose mutation has been reported to convey NFZ resistance are given as sticks with color-coding by elements (grey, carbon; blue, nitrogen; red, oxygen). Labels give the amino acid residue position in the immature protein from *Oryza sativa* (Acc. A2XDA1.2) including its N-terminal 87 amino acid transit peptide.
(DOCX)

S6 Fig Association with liposomal membranes and oligomeric assembly of Arg300Ser PDS. (A) SDS-PAGE analysis (12%, Coomassie-stained) of liposomal binding assays, carried out according to [6]. Lanes represent the liposome-bound PDS protein obtained from one PDS assay. WT, wild type OsPDS-His_6_. (B) Elution traces of wild type OsPDS-His_6_ and the mutant enzyme Arg300Ser monitored at 280 nm upon GPC analysis (Superose 6 10/300 GL column), carried out as reported previously [6]. The dominant high mass peak (oligo) represents the flavinylated and active PDS homooligomer, the low mass peaks represent the unflavinylated, inactive PDS monomer (mono) and free FAD that has been released from PDS upon sample handling and GPC analysis. The absence of peaks in the void volume (V_0_) indicates that higher order protein aggregates do not form.
(DOCX)

S1 Appendix Supplemental results. Dynamic modeling of PDS reaction time courses encompassing forward and reverse reactions.
(DOCX)

S2 Appendix Supplemental methods. Data preprocessing.
(DOCX)

## Abbreviations

CRTI: bacterial-type phytoene desaturase
CRTISO: carotene *cis-trans* isomerase
DPQ: decyl-plastoquinone
GPC: gel permeation chromatography
LCY: lycopene cyclase
MM: Michaelis-Menten
NFZ: norflurazon
p: phytoene
PDS: phytoene desaturase
pf: phytofluene
ZDS: ζ-carotene desaturase
ZISO: ζ-carotene *cis-trans* isomerase
z: ζ-carotene
