# Supplementary material for: Plant-type phytoene desaturase: Functional evaluation of structural implications
Source: PLoS One. 2017 Nov 27;12(11):e0187628. doi: 10.1371/journal.pone.0187628 (PMC5703498; doi:10.1371/journal.pone.0187628)
Supplement: S1 Fig — The PDS monomer has one long substrate channel with oxidized FAD near the bottom end. Phytoene is symmetric as indicated by the two arms (blue color and orange colors denote oxidized and reduced states, respectively). The carotene enters with one oxidized (saturated) end and is desaturated, thereby reducing FAD (ping). The resulting phytofluene retains one oxidized end and is expelled into the lipid phase. The channel can now be occupied by plastoquinone to oxidize FADred (pong) and to reconstitute the oxidized enzyme for a new round of catalysis. Because of this temporally separated succession of events, the two redox reactions are thought to be thermodynamically independent. The intermediate phytofluene, still possessing one half side being identical to that of phytoene, can as well be a PDS substrate by entering the substrate cavity with the saturated end. Increasing phytofluene amounts can therefore compete with phytoene for desaturation. (DOCX) [file pone.0187628.s001.docx]

**Figure S1. Postulated kinetic events during the ordered ping-pong bi-bi mechanism of PDS.** The PDS monomer has one long substrate channel with oxidized FAD near the bottom end. Phytoene is symmetric as indicated by the two arms (blue color and orange colors denote oxidized and reduced states, respectively). The carotene enters with one oxidized (saturated) end and is desaturated, thereby reducing FAD (ping). The resulting phytofluene retains one oxidized end and is expelled into the lipid phase. The channel can now be occupied by plastoquinone to oxidize FAD_red_ (pong) and to reconstitute the oxidized enzyme for a new round of catalysis. Because of this temporally separated succession of events, the two redox reactions are thought to be thermodynamically independent. The intermediate phytofluene, still possessing one half side being identical to that of phytoene, can as well be a PDS substrate by entering the substrate cavity with the saturated end. Increasing phytofluene amounts can therefore compete with phytoene for desaturation.
