# Supplementary material for: Plant-type phytoene desaturase: Functional evaluation of structural implications
Source: PLoS One. 2017 Nov 27;12(11):e0187628. doi: 10.1371/journal.pone.0187628 (PMC5703498; doi:10.1371/journal.pone.0187628)
Supplement: S2 Fig — 9,15,9’-tri-cis-ζ-carotene was purified from OsPDS-His6 assays (see Methods), photoisomerized to 9,9’-di-cis-ζ-carotene in day light and used as substrate with chromoplasts as described elsewhere [24]. The upper HPLC trace (HPLC system 4) represents a control assay incubated in the absence of the substrate showing background levels of prolycopene (1), proneurosporene (2) and of ζ-carotene isomers (3). The increased presence of (1) and (2) indicate the stereospecific identity of the 9,9’-di-cis-ζ-carotene added. The amount of ζ- (4) and β-carotene (5) present cannot change in aerobic assays [24] and therefore serve as an internal reference. The UV/VIS spectra of the substrate and the products are given as insets. (DOCX) [file pone.0187628.s002.docx]

**Figure S2. Conversion of 9,9’-di-*cis*-ζ-carotene by daffodil chromoplasts.**

9,15,9’-tri-*cis*-ζ-carotene was purified from OsPDS-His_6_ assays (see Methods), photoisomerized to 9,9’-di-*cis*-ζ-carotene in day light and used as substrate with chromoplasts as described elsewhere [24]. The upper HPLC trace (HPLC system 4) represents a control assay incubated in the absence of the substrate showing background levels of prolycopene (1), proneurosporene (2) and of ζ-carotene isomers (3). The increased presence of (1) and (2) indicate the stereospecific identity of the 9,9’-di-*cis*-ζ-carotene added. The amount of ζ- (4) and β-carotene (5) present cannot change in aerobic assays [24] and therefore serve as an internal reference. The UV/VIS spectra of the substrate and the products are given as insets.
