# Supplementary material for: Plant-type phytoene desaturase: Functional evaluation of structural implications
Source: PLoS One. 2017 Nov 27;12(11):e0187628. doi: 10.1371/journal.pone.0187628 (PMC5703498; doi:10.1371/journal.pone.0187628)
Supplement: S3 Fig — The profile likelihood, χ2, is plotted over a range of parameter values around the estimated optimal value marked by a dot. As reference, the 68% / 90% / 95% confidence level (CL) thresholds corresponding to χ2 = 1 / 2.71 / 3.84 are given as horizontal lines. (DOCX) [file pone.0187628.s003.docx]

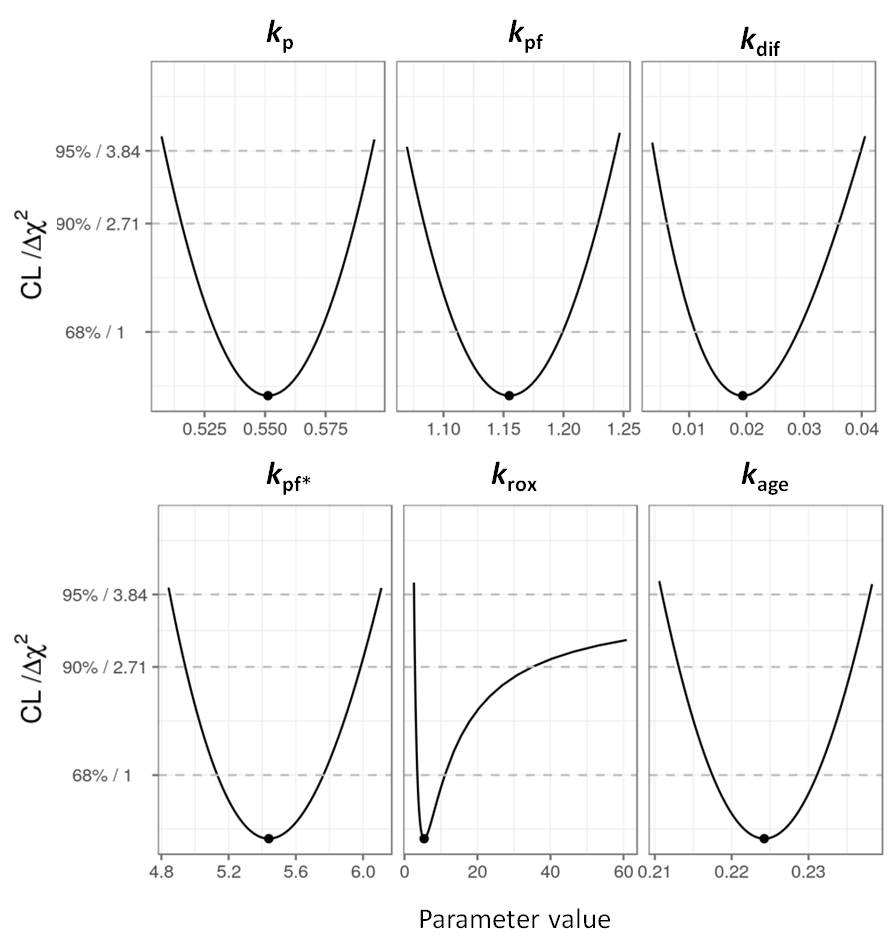


**Figure S3. Parameter likelihood profiles for the estimated dynamic parameters deduced from the substrate channeling model.**

The profile likelihood, χ^2^, is plotted over a range of parameter values around the estimated optimal value marked by a dot. As reference, the 68 % / 90 % / 95 % confidence level (CL) thresholds corresponding to χ^2^ = 1 / 2.71 / 3.84 are given as horizontal lines.
