# Supplementary material for: Plant-type phytoene desaturase: Functional evaluation of structural implications
Source: PLoS One. 2017 Nov 27;12(11):e0187628. doi: 10.1371/journal.pone.0187628 (PMC5703498; doi:10.1371/journal.pone.0187628)
Supplement: S4 Fig — The following residues are highlighted: 1, Phe162; 2, Arg300; 3, Tyr506; 4, Thr508 5, Leu538. Global sequence alignment was carried out with the Blosum62 matrix. Identical residues are green, similar residues greenish or yellow. Position numbering refers to the immature protein from O. sativa (A2XDA1.2) including its N-terminal 87 amino acid transit peptide. Organisms and accession numbers (from top to bottom): Oryza sativa, A2XDA1.2; Arabidopsis thaliana, Q07356.1; Chlorella zofingiensis, ABR20878.1; Hydrilla verticillata, AAT76434.1; Synechococcus elongatus PCC 7942, CAA39004.1; Synechocystis sp. PCC6803, CAA44452.1. (DOCX) [file pone.0187628.s004.docx]

**
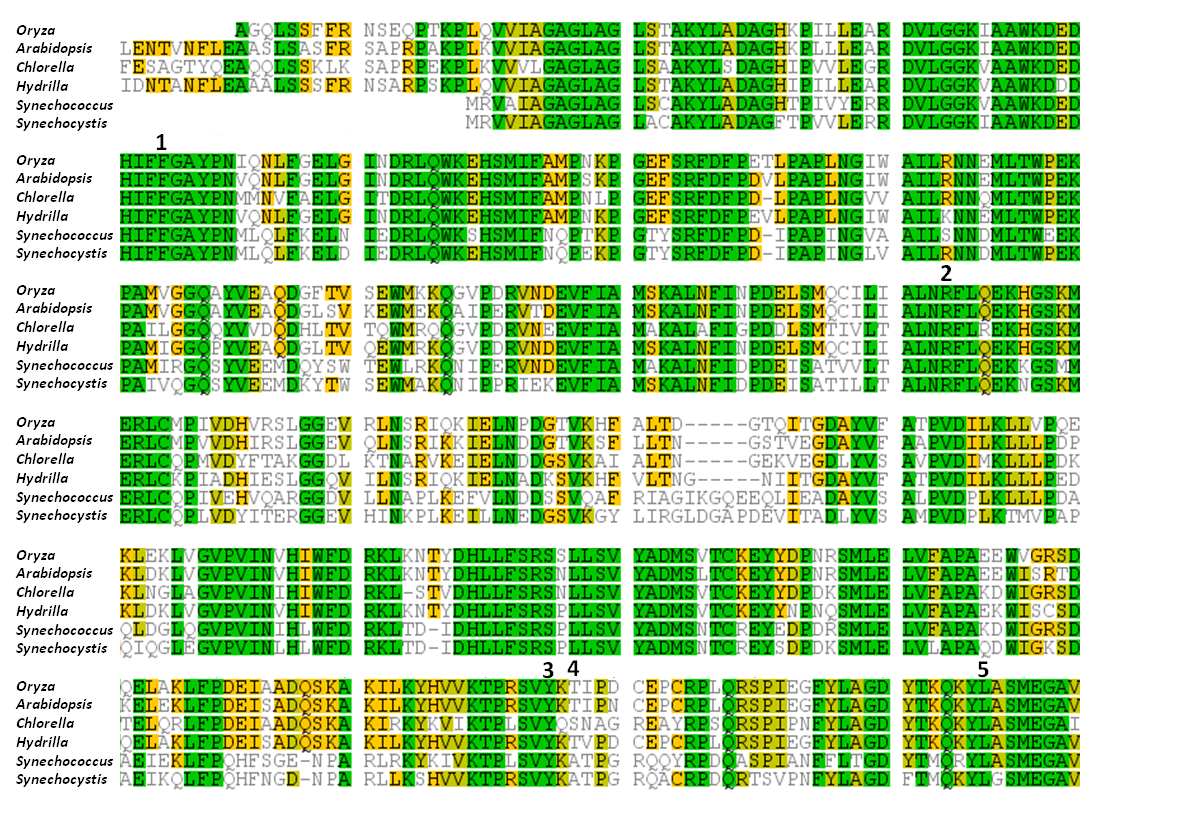
**

**Figure S4. Section of the protein alignment for PDS from *Oryza sativa* and cyanobacteria, algae and plants with reported mutations conferring NFZ resistance.**

The following residues are highlighted: 1, Phe_162_; 2, Arg_300_; 3, Tyr_506_; 4, Thr_508_ 5, Leu_538_. Global sequence alignment was carried out with the Blosum62 matrix. Identical residues are green, similar residues greenish or yellow. Position numbering refers to the immature protein from *O. sativa* (A2XDA1.2) including its N-terminal 87 amino acid transit peptide. Organisms and accession numbers (from top to bottom): *Oryza sativa*, A2XDA1.2; *Arabidopsis thaliana*, Q07356.1; *Chlorella zofingiensis*, ABR20878.1; *Hydrilla verticillata*, AAT76434.1; *Synechococcus elongatus* PCC 7942, CAA39004.1; *Synechocystis sp.* PCC6803, CAA44452.1.
