# Supplementary material for: Plant-type phytoene desaturase: Functional evaluation of structural implications
Source: PLoS One. 2017 Nov 27;12(11):e0187628. doi: 10.1371/journal.pone.0187628 (PMC5703498; doi:10.1371/journal.pone.0187628)
Supplement: S5 Fig — The inner surface of the PDS substrate cavity is depicted. The substrate cavity entry in the membrane binding domain is indicated by an arrow. The redox cofactor FAD is given as sticks representation in orange. Conserved residues whose mutation has been reported to convey NFZ resistance are given as sticks with color-coding by elements (grey, carbon; blue, nitrogen; red, oxygen). Labels give the amino acid residue position in the immature protein from Oryza sativa (Acc. A2XDA1.2) including its N-terminal 87 amino acid transit peptide. (DOCX) [file pone.0187628.s005.docx]

**Figure S5. Substrate cavity of OsPDS-His_6_ containing the redox cofactor FAD.**

The inner surface of the PDS substrate cavity is depicted. The substrate cavity entry in the membrane binding domain is indicated by an arrow. The redox cofactor FAD is given as sticks representation in orange. Conserved residues whose mutation has been reported to convey NFZ resistance are given as sticks with color-coding by elements (grey, carbon; blue, nitrogen; red, oxygen). Labels give the amino acid residue position in the immature protein from *Oryza sativa* (Acc. A2XDA1.2) including its N-terminal 87 amino acid transit peptide.
