# Supplementary material for: Plant-type phytoene desaturase: Functional evaluation of structural implications
Source: PLoS One. 2017 Nov 27;12(11):e0187628. doi: 10.1371/journal.pone.0187628 (PMC5703498; doi:10.1371/journal.pone.0187628)
Supplement: S6 Fig — (A) SDS-PAGE analysis (12%, Coomassie-stained) of liposomal binding assays, carried out according to [6]. Lanes represent the liposome-bound PDS protein obtained from one PDS assay. WT, wild type OsPDS-His6. (B) Elution traces of wild type OsPDS-His6 and the mutant enzyme Arg300Ser monitored at 280 nm upon GPC analysis (Superose 6 10/300 GL column), carried out as reported previously [6]. The dominant high mass peak (oligo) represents the flavinylated and active PDS homooligomer, the low mass peaks represent the unflavinylated, inactive PDS monomer (mono) and free FAD that has been released from PDS upon sample handling and GPC analysis. The absence of peaks in the void volume (V0) indicates that higher order protein aggregates do not form. (DOCX) [file pone.0187628.s006.docx]

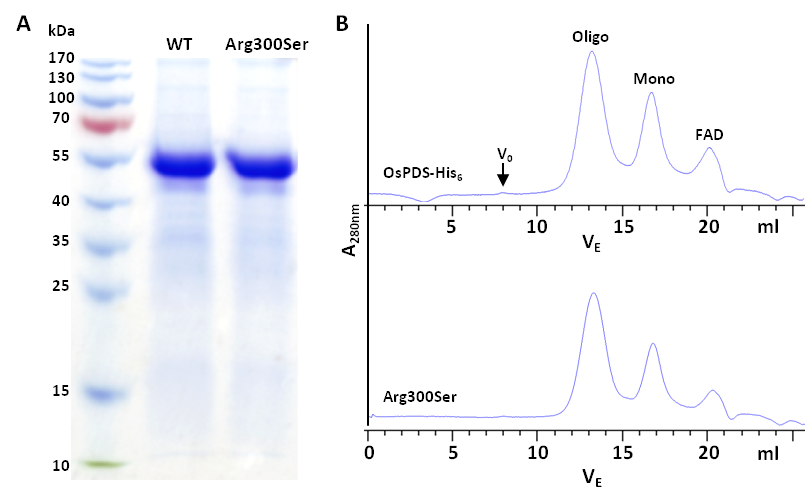


**Figure S6. Association with liposomal membranes and oligomeric assembly of Arg300Ser PDS.**

(A) SDS-PAGE analysis (12 %, Coomassie-stained) of liposomal binding assays, carried out according to [6]. Lanes represent the liposome-bound PDS protein obtained from one PDS assay. WT, wild type OsPDS-His_6_. (B) Elution traces of wild type OsPDS-His_6_ and the mutant enzyme Arg300Ser monitored at 280 nm upon GPC analysis (Superose 6 10/300 GL column), carried out as reported previously [6]. The dominant high mass peak (oligo) represents the flavinylated and active PDS homooligomer, the low mass peaks represent the unflavinylated, inactive PDS monomer (mono) and free FAD that has been released from PDS upon sample handling and GPC analysis. The absence of peaks in the void volume (V_0_) indicates that higher order protein aggregates do not form.
