# Supplementary material for: Plant-type phytoene desaturase: Functional evaluation of structural implications
Source: PLoS One. 2017 Nov 27;12(11):e0187628. doi: 10.1371/journal.pone.0187628 (PMC5703498; doi:10.1371/journal.pone.0187628)
Supplement: S1 Appendix — Dynamic modeling of PDS reaction time courses encompassing forward and reverse reactions. (DOCX) [file pone.0187628.s007.docx]

**Appendix S1**

**Supplemental results**

**Dynamic modeling of PDS reaction time courses encompassing forward and reverse reactions**

In this approach, all three main processes in PDS – namely phytoene desaturation (i), phytofluene desaturation (ii) and plastoquinone reduction (iii) – are implemented as forward and reverse reactions of the relevant sub-processes. These include enzyme-substrate association, the redox reaction of FAD and the substrate and enzyme-product dissociation.

1. p + FAD ⇌ p•FAD ⇌ pf•FADr ⇌ pf + FADr
2. pf + FAD ⇌ pf•FAD ⇌ z•FADr ⇌ z + FADr
3. DPQ + FADr ⇌ DPQ•FADr ⇌ DPQH_2_•FAD ⇌ DPQH_2_ + FAD

The resulting set of ordinary differential equations is given by:


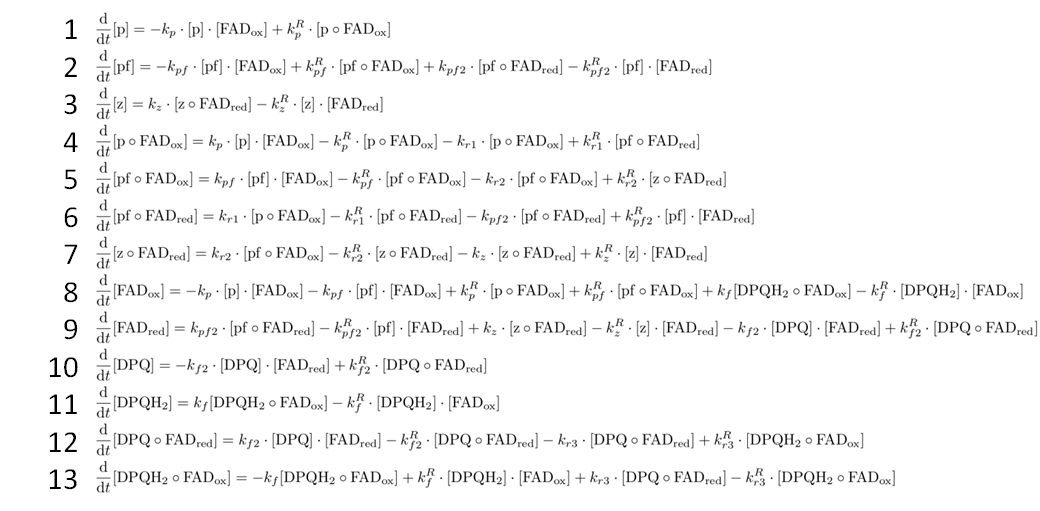


The resulting kinetic scheme is depicted in Fig. 1 A. In total the model contains 18 dynamic parameters (rate constants) in addition to the scaling parameters l_1_ and l_2_ (see Methods section) and one initial state parameter per condition. This model does not implement time-dependent enzyme inactivation and does not consider an effect of substrate channeling but assumes independent action of every monomeric subunit. By the large number of reversible reactions, the model allows for non-trivial steady states making it possible to describe the plateaus of the reaction time courses “p high” and “p low” and “pf“ upon individual parameter estimation (dashed lines in Fig. 1 B, C; “pf” not shown). However, any attempt to fit the reaction time courses using simultaneous parameter estimation fails even if using only “p high” and “p low” (solid lines in Fig. 1 B, C). The observed plateaus of pf and z formation cannot be explained by simply reaching steady state, indicating that an essential feature and process is missing in the model that is responsible for the ceasing of phytoene and phytofluene conversion by OsPDS-His_6_. Several mechanisms could lead to this behaviour, the simplest one being enzyme inactivation by denaturation. In accordance with this option, it was experimentally shown that the phytoene and phytofluene conversion could be restarted by adding fresh enzyme (see Fig. 2 D main text) but not by the addition of additional substrates (phytoene or DPQ). Therefore, the model was extended by a process describing the inactivation of the enzyme. Several mechanisms for the enzyme inactivation process of the oxidized and reduced state of the enzyme (FAD_ox_ and FAD_red_, respectively) were implemented and it was tested which implementation would allow describing the data upon simultaneous parameter estimation.

First, it turned out that enzyme inactivation is most likely not directly triggered by the enzymatic reactions, i.e. not proportional to the turnover number catalyzed by the enzyme, but is rather a time-dependent process as commonly encountered in enzymology with highly purified enzymes. This former case was implemented by linking the enzyme inactivation rate to the integrated turnover number of PDS, the latter case by a constant inactivation rate reducing the amount of the reduced and oxidized state of the enzyme over time. In order to further investigate the time-dependent PDS inactivation, it was tested whether separate inactivation rate constants for the FAD_ox_ and the FAD_red_ state of the enzyme would improve the fit results. Assuming separate rate constants, the parameter profiles in Fig. 2 reveal that the parameter for inactivation of the FAD_ox_ state (k_age_ FAD_ox_) is well determined whereas the parameter for inactivation of the FAD_red_ state (k_age_ FAD_ox_) is not. I.e., k_age_ FAD_red_ can be set to either zero or the same value as k_age_ FAD_ox_ without deteriorating the fit results, meaning that it cannot be determined whether inactivation of the reduced enzyme state is as fast as for the oxidized state or whether the reduced state is not inactivated at all. Considering that the enzyme inactivation is time-dependent but not dependent on enzymatic activity, it is feasible to assume that inactivation does not depend on the activity-related redox state of PDS either. Thus, it was decided to introduce a common rate constant, termed k_age_, for the inactivation of FAD_ox_ and FAD_red_ which decreased the amount of oxidized and reduced PDS over time – by extending equations 8 and 9 by the terms $-k_{\mathrm{age}}\cdot[\mathrm{FAD}_{\mathrm{ox}}]$ and $- k_{\mathrm{age}}\cdot\left[ \mathrm{FAD}_{\mathrm{red}} \right]$, respectively. The resulting comprehensive model encompassed 19 dynamic parameters, including 18 forward and reverse reactions of the PDS overall reaction and enzyme inactivation (Fig. 1 A). It allowed describing the phytoene reaction time courses “p high” and “p low” upon simultaneous parameter estimation (Fig. 1 D, E). The corresponding parameter likelihood profiles are given in Fig. 3.

**
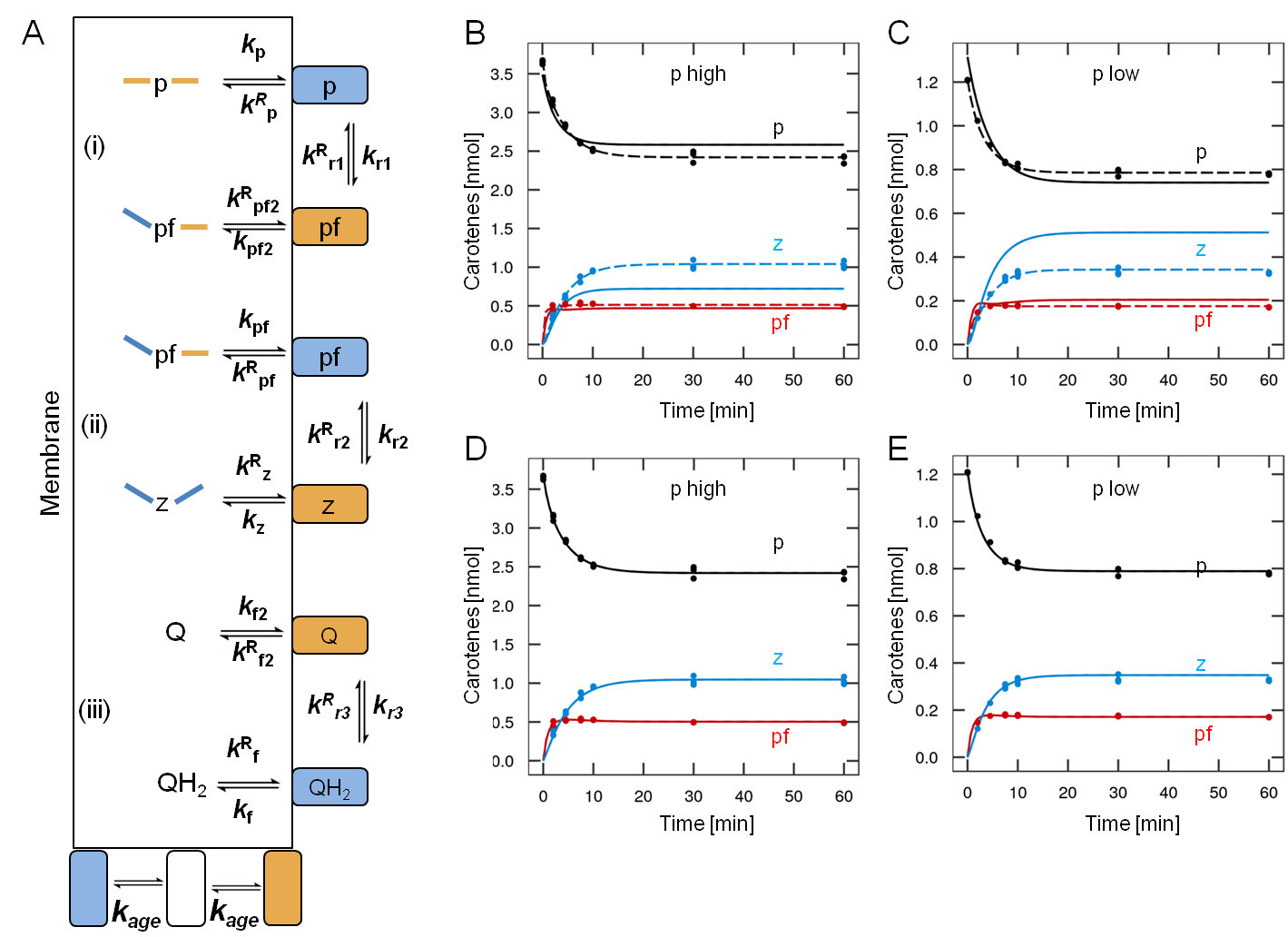
**

**Fig. 1 Kinetic scheme of the PDS reaction and dynamic modeling of PDS reaction time courses.**

(A) Kinetic scheme of the PDS reaction. PDS monomeric subunits (rectangles) within the homotetramer are assumed to work independently. Red/blue color denotes reduced/oxidized half sides of substrates phytoene (p), phytofluene (pf) and ζ-carotene (z) and the redox state of the PDS-bound FAD. The overall reaction comprises the three main processes phytoene desaturation (i), phytofluene desaturation (ii) and plastoquinone reduction (iii). Every process encompasses three equilibria indicated by the reaction arrows: association-dissociation of enzyme and substrate, desaturation-saturation of substrate and dissociation-association of enzyme and product. A rate constant was assigned to every forward (k_x_) and reverse reaction (k^R^_x_). All hydrophobic carotene substrates and decylplastoquinone (Q) are soluble in the hydrophobic core of liposomal membranes. (B-D) Observed data and dynamic modeling of PDS reaction time courses. Reaction time courses were conducted with 3.7 nmol phytoene (p high; B, D), and 1.3 nmol phytoene (p low; C, E). The observables are given as data points, measurements were carried out in triplicate (black, phytoene; red, phytofluene; blue, ζ-carotene). In B and C, the model fit is represented as dashed lines (individual parameter estimation) and as solid lines (simultaneous parameter estimation of both reaction time courses). In D and E, enzyme inactivation was included into the model and the model fit is given as solid lines.


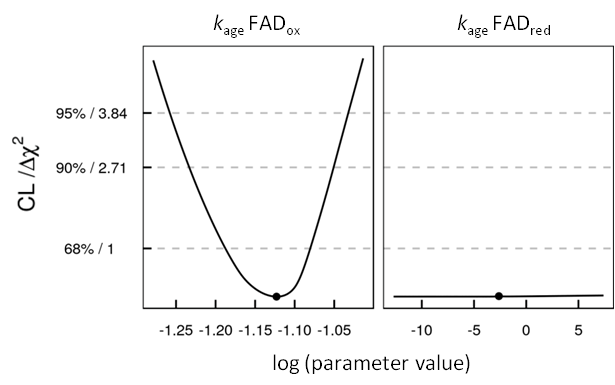


**Fig. 2. Likelihood profiles for the enzyme inactivation parameter k_age_ for oxidized and reduced PDS.**

The profile likelihood, Δχ^2^, is plotted over a range of logarithmic parameter values around the estimated optimal value marked by a dot. As reference, the 68 % / 90 % / 95 % confidence level (CL) thresholds corresponding to Δχ^2^ = 1 / 2.71 / 3.84 are given as horizontal lines.

The likelihood profiles revealed that only two of the 19 dynamic parameters, namely k_age_ and k_z_, can be identified from the available data. All other parameters can be varied across a wide range of parameter values without impairing the goodness of fit, i.e. the χ^2^ value for the fit. This deficiency is denoted as overparameterization, meaning that not all parameters could be determined with the available data. Overparametrization can be overcome by reducing model complexity to the relevant processes that can be inferred from the data. Some processes may be directly discarded from the model if the corresponding parameter value is in accordance with zero and does not couple to other parameters. If the parameters are coupled, meaning that adjustment of other parameters can compensate the change of the parameter of interest, a more sophisticated approach for model reduction is necessary. For the model presented in Fig. 1 it became apparent that not all sub-processes for each of the main processes (i) to (iii) – namely phytoene desaturation, phytofluene desaturation and plastoquionone reduction – can be dissolved based on the time course data. Therefore, model reductions were tested combining several sub-processes into effective processes. The model resulting from this model reduction process is shown in Fig. 4 A in the main text and was the basis for further modeling efforts.


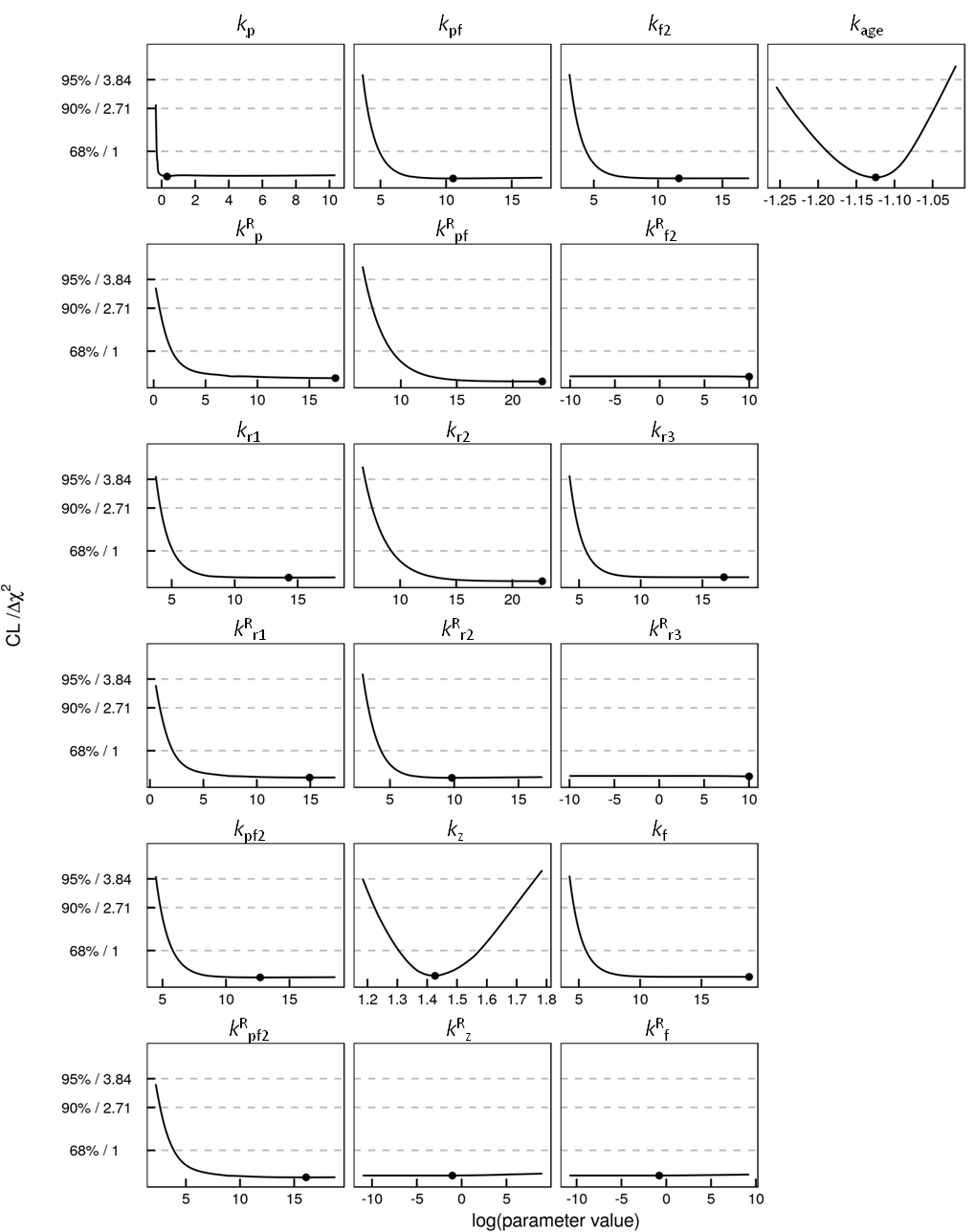


**Fig. 3. Parameter likelihood profiles for the comprehensive PDS model.**

The comprehensive PDS model, including enzyme inactivation with k_age_, is depicted in Fig. 1 A and was used to model the phytoene conversion reaction time courses “p high” and “p low”. The profile likelihood, Δχ^2^, is plotted over a range of logarithmic parameter values around the estimated optimal value marked by a dot. As reference, the 68 % / 90 % / 95 % confidence level (CL) thresholds corresponding to Δχ^2^ = 1 / 2.71 / 3.84 are given as horizontal lines.
