# Supplementary material for: Plant-type phytoene desaturase: Functional evaluation of structural implications
Source: PLoS One. 2017 Nov 27;12(11):e0187628. doi: 10.1371/journal.pone.0187628 (PMC5703498; doi:10.1371/journal.pone.0187628)
Supplement: S2 Appendix — Data preprocessing. (DOCX) [file pone.0187628.s008.docx]

**Appendix S2**

**Supplemental Methods**

**Data preprocessing**

Measurement uncertainties were estimated from the empirical variances via a maximum likelihood method, assuming the error model
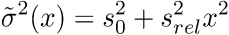
 where x is the measurement value. The parameters
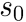
 and
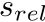
 for the absolute and relative uncertainty contribution were obtained by minimizing the negative log-likelihood
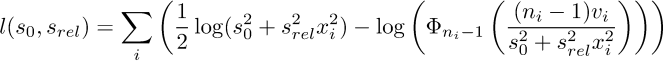
, where
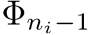
 is the probability density of the
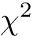
-distribution with n_i_ - 1 degrees of freedom and the index i corresponds to data point i with mean value x_i_ and empirical variance υ_i_ determined from n_i_ replicates. Individual
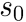
 and
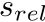
 parameters were calculated per reaction time course and observable. Finally, the standard error of the mean
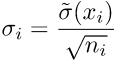
 was computed from the estimated standard deviation
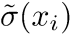
. The variances as a function of the corresponding mean values are shown in Fig. 1 together with the calibrated error model. The error model parameter values are given in Tab. 1.

**Tab. 1. Parameters of the error model.**

| observable | 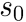 | 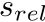 |
| --- | --- | --- |
| p | 1.34e^-6^ | 0.015 |
| pf | 7.75e^-7^ | 0.033 |
| z | 0.012 | 0.036 |


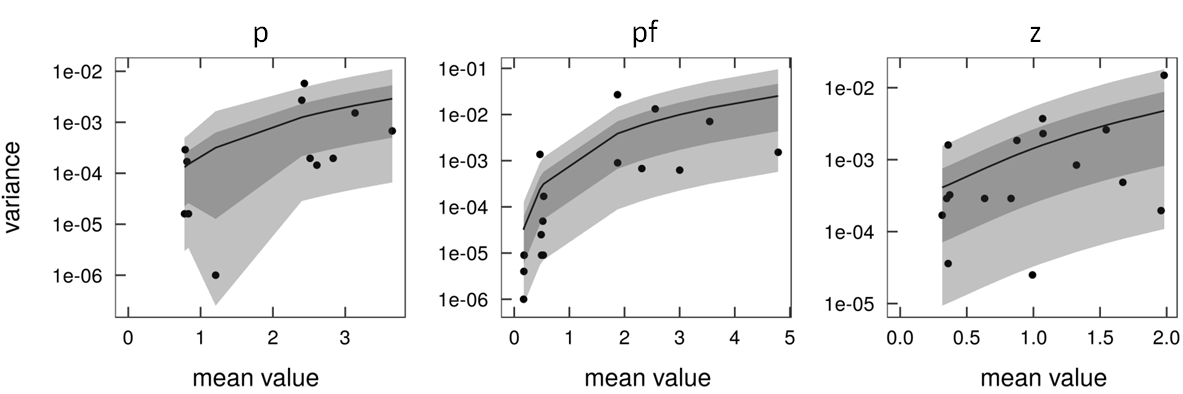


**Fig. 1. Variances versus mean values.**

The empirical mean values (n = 3) for p, pf and z (from left to right panel) obtained from the reaction time courses are given as dots plotted against the variance. The fitted error model is given as solid line, with bright grey and dark grey shadowed areas representing the 68 % and 95 % uncertainty bands of the variance values, respectively. They account for the number of replicates from which the variance was estimated.

In Fig. 2, the total sum of carotenes is shown for each dataset. Besides expected fluctuations, a systematic time-dependent variation is visible. This is because of the conversion of 15-*cis*-pyhtoene into 9,15-di-*cis*-phytofluene and 9,15,9’-tri-*cis*-ζ-carotene. The molar extinction coefficient is only reported for the former, whereas the molar extinction coefficients for phytofluene and ζ-carotene are only known for the all-*trans* species – this leading to inaccuracies during their quantification. To account for this, scaling factors producing a constant carotene sum were obtained by an optimization routine, described in the methods section. The result of the optimization is shown in Fig. 2. As expected, a flat course is obtained for each reaction time course still showing random but not systematic fluctuations. The profile likelihood for the scaling parameters l_1_ and l_2_ is shown in Fig. 3 and the corresponding optimal values are l_1_ = 1.06 ± 0.04 and l_2_ = 0.61 ± 0.03.


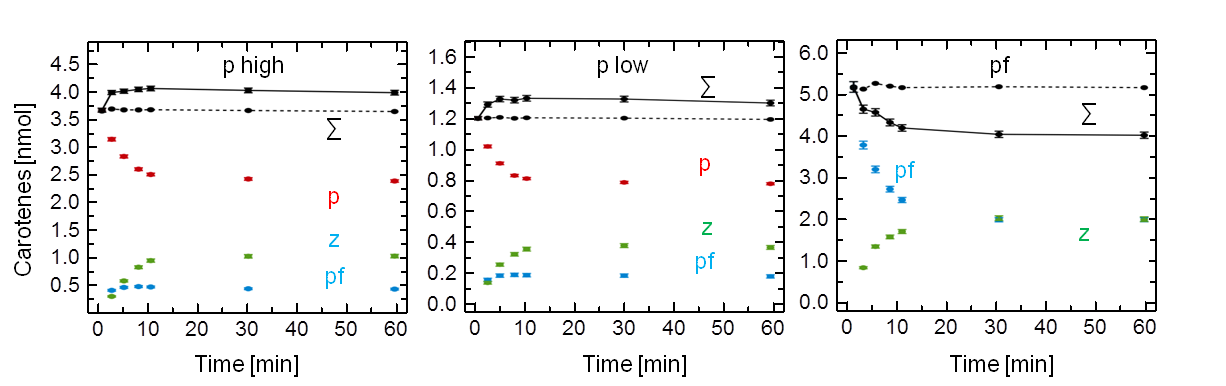


**Fig. 2. Carotene sum during PDS reaction time course.**

The measured amounts of p, pf and z are given as data points as mean values ± SEM (n=3). p, red; pf, blue; green, z. The derived carotene sum is given as black data point representing mean values ± SEM and trend lines for the carotene sum ∑ are given without scaling (solid line) or upon use of the scaling model (dashed line). The scaling model accounts for inaccuracies during carotene quantification due to lack of knowledge about molar extinction coefficients for poly-*cis*-carotenes. The value of the first data point was used as constant in the optimization.


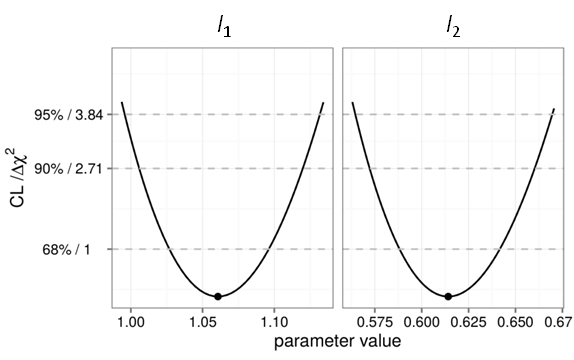


**Fig. 3. Likelihood profiles for the *l*_1_ and *l*_2_ parameters obtained for the scaling model.**

The profile likelihood, Δχ^2^, is plotted over a range of logarithmic parameter values around the estimated optimal value marked by a dot. As reference, the 68 % / 90 % / 95 % confidence level (CL) thresholds corresponding to Δχ^2^ = 1 / 2.71 / 3.84 are given as horizontal lines.
